# The chemokine receptor CCR10 promotes inflammation-driven hepatocarcinogenesis via PI3K/Akt pathway activation

**DOI:** 10.1038/s41419-018-0267-9

**Published:** 2018-02-14

**Authors:** Qiong Wu, Jin-xian Chen, Yu Chen, Li-li Cai, Xiao-zhong Wang, Wu-hua Guo, Jian-feng Zheng

**Affiliations:** 1 Department of Clinical Laboratory Medicine, The Second Affiliated Hospital of Nanchang University, Nanchang, 330006 China; 2 Department of Gastrointestinal Surgery, Renji Hospital, Shanghai Jiao Tong University School of Medicine, Shanghai, 200127 China; 3 Department of Gastroenterology, The Second Affiliated Hospital of Nanchang University, Nanchang, 330006 China; 4 Department of Laboratory Medicine, Affiliated Hospital of Guilin Medical University, Guilin, 541001 China

## Abstract

G-protein-coupled receptor (GPCR)-related proteins are dysregulated and the GPCR CC-chemokine receptor 10 (CCR10) is significantly upregulated in inflammation-driven HCC. However, CCR10′s role in inflammation-driven hepatocarcinogenesis remains unknown. The aim of this study was to evaluate the role of CCR10 in inflammation-driven hepatocarcinogenesis. Via a targeted gene expression microarray screening alterations in GPCR family gene expression, we found CCR10 to be significantly upregulated in hepatocytes isolated from inflammation-driven human HCC tumors and matching paracancerous tissues. Tetrachloromethane (CCl4)-induced and diethylnitrosamine (DEN)-induced murine models of inflammatory hepatocarcinogenesis displayed significant hepatocellular TNF and CCR10 upregulation. Exogenous TNF applied to HepG2 and LO2 cell lines as well as wild-type (WT) mice significantly upregulated hepatocellular CCR10 expression, Akt phosphorylation, PCNA expression, and hepatocellular proliferation. Additionally, exogenous TNF significantly upregulated secretion of the natural CCR10 ligand-agonist CCL28 from both cell lines. Transgenic CCR10-knockout (CCR10 KO) in DEN-treated mice significantly increased hepatocellular apoptosis levels and significantly lowered compensatory hepatocellular proliferation but did not affect upstream TNF expression. In addition, DEN-treated CCR10 KO mice showed a significantly lower liver weight/body weight ratio, significantly lower liver tumor incidence, and significantly smaller tumors. Moreover, exogenous CCR10 expression significantly raised xenograft tumor growth in Balb/c nude mice. In vitro, CCR10 transfection or CCL28 treatment in HepG2 and LO2 cell lines significantly increased Akt phosphorylation, PCNA expression, and cell proliferation, while CCR10 silencing or Akt inhibition produced the opposite effects. In vivo, hepatocytes isolated from HCC tumor tissue and matching paracancerous tissue in DEN-treated CCR10 KO mice showed significantly lower Akt phosphorylation and PCNA expression relative to WT hepatocytes. In conclusion, inflammation-induced TNF promotes hepatocellular CCR10 expression and downstream PI3K/Akt-mediated hepatocarcinogenesis. CCR10 appears to function as a linkage between TNF stimulation and downstream PI3K/Akt pathway activation and shows promise as a potential therapeutic target for inflammation-driven HCC.

## Introduction

Hepatocellular carcinoma (HCC), the most common liver cancer, is a leading cause of cancer mortality globally^1^. Hepatocarcinogenesis displays strong linkages with underlying chronic inflammatory processes in liver parenchyma, such as chronic viral infection (e.g., hepatitis B virus (HBV), hepatitis C virus), non-alcoholic fatty liver disease (NAFLD), alcoholism, and aflatoxin exposure^2^. These chronic inflammatory processes are initiated by Kupffer cell activation, resulting in secretion of pro-inflammatory cytokines, such as tumor necrosis factor (TNF), interleukin-1β (IL-1β) and interleukin-6 (IL-6)^3^. These pro-inflammatory cytokines create the microenvironment for hepatocarcinogenesis through promoting pre-malignant cell proliferation, malignant tumor growth, angiogenesis, and metastasis^3^.

Although the precise mechanism(s) by which TNF promotes hepatocarcinogenesis still remains unclear, several G-protein-coupled receptors (GPCRs) have been linked to regulating TNF-driven inflammatory processes^4^. GPCRs are the largest superfamily of cell-surface receptor complexes and uniformly consist of a seven transmembrane-helical receptor domain, a trimeric αβγ G-protein, and an effector molecule that transduces the receptor-initiated signal into the cytoplasm^5^. Various GPCRs are upregulated in HCC as well as other human cancers^5^, yet the exact mechanism(s) by which these GPCR-related changes promote inflammation-driven hepatocarcinogenesis remain unclear^6^.

One type of GPCR, CC-chemokine receptors, are upregulated in various kinds of cancer cells in reaction to pro-inflammatory cytokines^7^. Yang et al.’s recent work revealed that one CC-chemokine receptor in particular, CC-chemokine receptor 10 (CCR10, GPR2), is significantly upregulated in inflammation-driven HCC tumors. However, no study to date has examined the role that CCR10 may play in inflammation-driven HCC. Therefore, the aim of this study will be to evaluate the role of CCR10 in inflammation-driven hepatocarcinogenesis.

## Materials and methods

### Ethics and consent

The clinical protocols of this study were approved by the Ethics Committee of The Second Affiliated Hospital of Nanchang University (Nanchang, China) and the Ethics Committee of Renji Hospital (Shanghai Jiao Tong University School of Medicine, Shanghai, China). Written informed consent was obtained from each participant prior to inclusion in this study. All animal procedures were approved by the Animal Care and Use Committee at Renji Hospital (Shanghai Jiao Tong University School of Medicine).

### Patient recruitment and liver tissue specimens

HBV-infected HCC patients and hepatic hemangioma patients were recruited for this study. The inclusion criteria for HCC patients were: (i) aged 18–75 years, (ii) hepatitis B surface antigen-positive, or HBsAg-negative and HBV DNA-positive, (iii) Child-Pugh score of 5–6, (iv) presence of at least one resectable hepatic tumor, and (v) definitive HCC diagnosis based on postoperative histopathological analysis. The exclusion criteria for HCC patients were: (i) history of antiviral, radiotherapy, or transcatheter arterial chemoembolization (TACE) therapy; (ii) infection with hepatitis C, hepatitis D, or human immunodeficiency viruses; (iii) presence of other malignant tumors, (iv) presence of extra-hepatic metastases; (v) history of drug abuse, (vi) pregnancy; or (vii) presence of immunocompromising disease.

The inclusion criteria for hepatic hemangioma patients were: (i) aged 18–75 years, (ii) Child-Pugh score of 5–6, (iii) presence of at least one resectable hepatic tumor, and (iv) hepatic hemangioma diagnosis based on postoperative histopathological analysis. The exclusion criteria for hepatic hemangioma patients were: (i) history of antiviral, radiotherapy, or TACE therapy; (ii) infection with hepatitis B, hepatitis C, hepatitis D, or human immunodeficiency viruses; (iii) presence of cirrhosis or hepatitis, (iv) presence of other tumors, (v) history of drug abuse, (vi) pregnancy; or (vii) presence of immunocompromising disease.

Paired liver tissue specimens from HCC tumors and paracancerous tissues were surgically extracted from HBV-infected HCC participants (*n* = 81). Healthy liver tissue specimens were surgically extracted from hepatic hemangioma patients for use as normal controls (*n* = 28). Demographic and clinical characteristics are detailed in Supplementary Table 1 (Online Supplementary Materials).

### Hepatocyte cell lines

HepG2, Hep3B, and LO2 cell lines were purchased from the Shanghai Institute of Cell Biology (Chinese Academy of Sciences, Shanghai, China). Cell lines were tested for mycoplasma contamination prior to culture. HepG2 and Hep3B cells were cultured in Dulbecco’s Modified Eagle’s Medium, while LO2 cells were cultured in RPMI-1640 medium plus with 10% fetal bovine serum (all Gibco BRL, Grand Island, NY, USA). For some in vitro experiments, HepG2 and LO2 cell lines were pre-treated with the pro-inflammatory cytokine TNF (concentrations as indicated; R&D Systems, Minneapolis, MN, USA) for 4 h, recombinant human CC chemokine ligand 28 (CCL28) (400 nM; R&D Systems) for 2 h, or the allosteric Akt inhibitor A6730 (10 μM; Sigma, St. Louis, MO, USA) for 2 h^8^. Human CCL28 is a natural ligand-agonist for human CCR10^9^. A6730 produces a conformational change in Akt that prevents it from associating with upstream kinases, thereby abrogating Akt phosphorylation^10^.

### Mice subjects and ccr10 knockout construction

Male C57BL/6 mice were purchased from the Shanghai Laboratory Animal Center (Chinese Academy of Sciences). CCR10+/+ wild-type (WT) and CCR10−/− knockout (CCR10 KO) litter mates on a C57BL/6 background were purchased from Shanghai Genechem Company Ltd. (Shanghai, China). Construction of CCR10 KO mice was performed according to Jin et al.’s well-established protocol (Supplementary Figure 1A, Online Supplementary Materials)^11^. Briefly, a targeting construct consisting of a 3.6-kb fragment exactly 5′ of the CCR10 start codon (the 5′ arm), followed by the EGFP coding sequence, followed by the loxP-flanked neo cassette, and followed by a 0.5-kb fragment containing a 0.2-kb 3′ portion of CCR10 as well as a 0.3-kb 3′ noncoding region (the 3′ arm). The 3.6-kb 5′ arm and the 0.5-kb 3′ arm were amplified by PCR from murine genomic DNA and sequence confirmed. Then, the linearized targeting construct was transfected into the J1 murine embryonic stem cell line, of which G418/gancyclovir-resistant clones were PCR-screened for KO recombinants with a primer set amplifying a 0.9-kb fragment from the 3′ end of CCR10 (Supplementary Figure 1B, Online Supplementary Materials). The targeted allele (neo+) replaces the 1.7-kb CCR10 fragment (encoding for the N-terminal extracellular domain and the seven transmembrane domains of CCR10) with a loxP-flanked neo cassette and an EGFP sequence. The resulting neo + CCR10-KO clones were validated by Southern blotting with a probe specific for a region 5′ upstream from the target (Supplementary Figure 1C, Online Supplementary Materials). KO clones were then microinjected into blastocysts from C57BL/6 (B6) mice to generate chimeras. Male chimeras were then cross-bred with EIIa-Cre transgenic females to produce heterozygous CCR10-KO/EGFP-knock-in mice. These heterozygous mice were then backcrossed to wild-type B6 mice over nine generations and intercrossed to produce homozygous CCR10-KO/EGFP-knock-in mice (Supplementary Figure 1D, Online Supplementary Materials). Normal hepatocyte progenitor development in fetal livers of homozygous and heterozygous CCR10-KO mice as well as EGFP expression on gated CD117+ /CD34+ hepatocyte progenitor cells from homozygous and heterozygous CCR10-KO mice were validated prior to experimentation (Supplementary Figure 1E-F, Online Supplementary Materials)^11,12^. Moreover, results of liver function testing were statistically equivalent between the WT and CCR10-KO groups (Supplementary Table 2, Online Supplementary Materials)^13^.

In addition, male Balb/c athymic nude mice were purchased from the Animal Center at Shanghai Laboratory Animal Center (Chinese Academy of Sciences, Shanghai, China). All mice were held in pathogen-free, micro-isolator cages on a 12 h/12 h light/dark cycle. All mice were fed standard chow and water ad libitum.

### Gene microarray

Total RNA from isolated hepatocytes was extracted with a TRIzol reagent (Invitrogen, Carlsbad, CA, USA) and then purified using a mirVana miRNA Isolation Kit (Ambion, Austin, TX, USA). RNA purities and concentrations were spectrophotometrically assessed using OD260/280 absorbance readings from a NanoDrop ND-1000 (NanoDrop Technologies, Wilmington, DE, USA). Fluorescently-labeled cDNA (Cy3-dCTP) was synthesized with Eberwine’s linear RNA amplification method using a cRNA Amplification and Labelling Kit (CapitalBio, Beijing, China) followed by enzymatic reaction. Double-stranded cDNA (dsDNA) was synthesized and then purified with a NucleoSpin Extract II Kit (Macherey-Nagel, Düren, Germany). The resulting dsDNA was eluted in elution buffer (total volume of 30 μl), vacuum-evaporated to a total volume of 16 μl, and in vitro transcribed with a T7 Enzyme Mix (Takara, Dalian, China) in a total reaction volume of 40 μl at 37 °C for 14 h. CbcScript II reverse transcriptase was used for reverse transcription followed by Klenow enzyme labeling.

A gene microarray was performed with a SurePrint G3 Human Gene Expression 8 × 60 K Array (Agilent Technologies, Santa Clara, CA, USA). There were eight identical arrays per slide. Each array consisted of 27958 Entrez Gene RNA probes as well as 1280 Agilent control probes (www.chem.agilent.com/ library/brochures/5989–3805EN.pdf). An Agilent hybridization oven was used to perform overnight hybridization (20 RPM, 42 °C). The array was consecutively rinsed with two solutions: the first solution was 0.2% SDS, 2 × SSC, (5 min, 42 °C), and the second solution was 0.2 × SSC (5 min, room temperature).

GeneSpring version 12 (Agilent) was used to analyze array data. Differentially-expressed genes were defined as those with a greater than 1.5 × absolute fold change and a *p*-value of less than 0.05 (post-Benjamini-Hochberg correction). The “Adjust Data” function of CLUSTER 3.0 was used to log_2_ transform and median center the array data. We then performed hierarchical clustering using average linkage. Java Treeview (Stanford University School of Medicine, Stanford, CA, USA) was used to perform tree visualization. The microarray data has been deposited to the Gene Expression Omnibus database under the accession number GSE95698.

### Construction of murine models

A tetrachloromethane (CCl_4_)-induced model of cirrhosis was constructed by i.p. injection of 20% CCl_4_ in olive oil (5 ml/kg, Sinopharm Chemical Reagent, China) into mice at 8 weeks of age for an 8-week period twice weekly. Vehicle control mice were i.p. injected with matching volumes of olive oil following the same regimen.

The short-term DEN-induced model of liver inflammation was constructed by a single i.p. injection of DEN in physiological saline (100 mg/kg) into mice at eight to 12 weeks of age. All mice were sacrificed after 10 days on a standard chow diet. TNF treatment was performed through two i.p. injections of TNF (40 μg/kg) 6 h prior to sacrifice. For in vivo experiments, recombinant murine CCL28 (R&D Systems) or the Akt inhibitor A6730 was i.p. injected (both 50 mg/kg) 6 h prior to sacrifice. Murine CCL28 is a natural ligand-agonist for murine CCR10 ^14^. Vehicle control mice were i.p. injected with matching volumes of physiological saline following the same regimen.

The long-term diethylnitrosamine (DEN)-induced model of inflammation-driven hepatocarcinogenesis was constructed by a single intraperitoneal (i.p.) injection of DEN in physiological saline (15 mg/kg) into mice at 15 days of age. All mice were sacrificed after 9 months on a standard chow diet. After sacrifice, all surface liver tumor nodules were counted and measured via a caliper.

### Hepatocyte isolation from liver tissue samples

Isolation of hepatocytes from liver tissue samples was performed by two-step collagenase isolation as previously described by LeCluyse et al.^15^ followed by fluorescence-activated cell sorting (FACS). Two-step collagenase-isolated hepatocytes were centrifuged down at 75 g for 5 min. The pellet was rinsed with Dulbecco’s PBS and frozen at −80 °C until the FACS procedure.

FACS was then applied to sort hepatocytes with a FITC-conjugated anti-human CD95 antibody (Ancell Corporation, Bayport, MN, USA) and a biotin-conjugated anti-human CD45 antibody (BD Pharmingen, San Diego, CA, USA) as previously described with minor modifications^16^. As CD95 is solely expressed by hepatocytes among all liver cell types, the morphological hepatocyte gate was defined by size, which was confirmed by a high frequency of CD95-expressing cells. To eliminate any immune cell contamination within the hepatocyte gate, we also applied sorting to eliminate cells expressing the immune cell marker CD45. After selecting for CD95+CD45- hepatocytes, we then comparatively assessed hepatocyte CCR10 expression with a phycoerythrin-conjugated anti-human CCR10 antibody (R&D Systems, Minneapolis, MN, USA).

### Real-time polymerase chain reaction (PCR)

Total RNA was extracted with a TRIzol reagent (Invitrogen). First-strand cDNA was synthesized from RNA with Superscript Reverse Transcriptase (Invitrogen). Real-time PCR was conducted using iQ SYBR Green Supermix (Bio-Rad, Hercules, CA, USA) and gene-specific primers on an ABI 7500 instrument (Applied Biosystems, Foster City, CA, USA). Real-time reverse transcriptase PCR (RT-PCR) was conducted using SYBR Green Master Mix (Invitrogen) on an ABI 7500 instrument (Applied Biosystems). Primer sequences are detailed in Supplementary Table 3 (Online Supplementary Materials). β-actin was used as an internal control for all PCR experiments.

Total liver samples from the experimental groups (e.g., HCC, CCl_4_, DEN, etc.) were used as positive controls for CD45 PCR assays.

### Western blotting

Western blotting was conducted with the following primary antibodies (all diluted 1:1000): anti-CCR10 (#sc-365957, Santa Cruz Biotechnology, Santa Cruz, CA, USA), anti-GFP (#sc-8334, Santa Cruz Biotechnology), anti-TNF (#3707, Cell Signaling Technology, Danvers, MA, USA), anti-PI3K (#SAB4502195, Sigma-Aldrich, St Louis, MO, USA),anti-Akt (#9272, Cell Signaling Technology), anti-phospho-Akt^Ser473^ (p-Akt) (#4060, Cell Signaling Technology), anti-cleaved caspase-3 (#9661, Cell Signaling Technology), anti-cleaved PARP (#9546, Cell Signaling Technology), anti-PCNA (#sc-56, Santa Cruz Biotechnology), and β-actin (#5125, Cell Signaling Technology). Bands were detected with ECL reagents (KeyGen Biotech, Nanjing, China). Band signals were quantified through densitometry. Results were reported as the ratio of the target protein’s densitometry units to β-actin’s densitometry units.

### Immunostaining

Liver sections were deparaffinized, rehydrated, and placed in 3% H_2_O_2_. Sections were then boiled in a Tris/EDTA-based antigen retrieval solution (pH 9.0) for 2 min. Then, goat serum (15%) was applied for 30 min to block non-specific antibody binding. For tissue specimen staining, slides were incubated with the following primary antibodies (all diluted 1:200): anti-CD68 (#sc-20060, Santa Cruz Biotechnology), anti-MPO (#sc-59600, Santa Cruz Biotechnology), and anti-Ki-67 (#sc-15402, Santa Cruz Biotechnology). Apoptosis levels were assayed via a terminal deoxynucleotidyl transferase-mediated dUTP-digoxigenin nick end labelling (TUNEL) assay kit (Biyuntian, Haimen, China). Cells with nuclei staining brown-to-black were deemed TUNEL + apoptotic cells. One thousand cells were counted in each specimen in order to calculate a TUNEL + percentage (%).

### Small Interfering RNA (siRNA) Silencing of CCR10 Expression

In vitro silencing of CCR10 via siRNA was conducted as previously described by Chen et al.^17^. The CCR10 siRNA targeting CCR10 mRNA’s coding region was constructed through BLOCK-iT RNAi Designer (Invitrogen) with a sense strand of GCUGGAUACUGCCGAUCUA and an anti-sense strand of UAGAUCGGCAGUAUCCAGC. Cells were seeded and cultured in six-well plates. Cells were then transfected with the CCR10 siRNA or a scrambled oligonucleotide negative control siRNA (sense: 5′-UUCUCCGAACGUGUCACGUTT-3′; anti-sense: 5′-ACGUGACACGUUCGGAGAATT-3′) using Lipofectamine 2000 (Invitrogen).All experiments were performed in triplicate wells and repeated in triplicate.

### Cell proliferation assay

Cell proliferation was assayed with a Cell Count Kit-8 (CCK-8, Beyotime, Haimen, China). Briefly, using a 96-well plate, the parent cell lines (1000 cells/well) were transfected with pcDNA3.0-EGFP control vector or pcDNA3.0-CCR10-EGFP vector or transfected with a CCR10 siRNA or a control siRNA (pcDNA3.0-EGFP vector was a gift from Doug Golenbock [Addgene plasmid # 13031]). Untreated parent cell lines as well as transfected cell lines were incubated for 24 h to 96 h. Then, a WST-8 solution was added to the culture (20 μl in 180 μl medium), left for 3 h at 37 °C, and 450-nm absorbance readings were collected.

### MPO activity assay

Liver tissue specimens were homogenized in 0.5% hexadecyltrimethylammonium bromide in phosphate buffer (50 mM, pH 6.0). Homogenates were then sonicated for 10 s, freeze-thawed thrice, and centrifuged for 15 min at 15,000 RPM. The resulting supernatant (100 μl) was mixed in O-dianisidine hydrochloride (0.167 mg/ml) and 0.0005% H_2_O_2_. MPO activity was spectrophotometrically assessed at 460-nm absorbance.

### Xenograft tumor construction in athymic nude mice

Cultured Hep3B cells were stably transfected with a pcDNA3.0-CCR10-HA overexpression vector or a pcDNA3.0-HA null vector. To construct the xenograft tumor model, Hep3B cells (5 × 10^6^ cells) were subcutaneously injected into flanks of athymic nude mice bilaterally at 6 weeks of age. Post-injection, calipers were used to monitor tumor growth at 3-day intervals over the course of 24 days. Tumor volumes were calculated as follows: tumor volume = 0.5 × tumor length × tumor width^2^.

### Quantification of supernatant CCL27 and CCL28

To quantify supernatant concentrations of the CCR10 ligands CCL27 and CCL28 in cultured cells, ELISA kits for human CCL27 and CCL28 were employed according to the manufacturer’s instructions (R&D Systems).

### Statistical analysis

Statistical analysis was conducted with GraphPad Prism and GraphPad InStat software version 6 (GraphPad Software, La Jolla, CA, USA). Results are reported as means ± standard deviations. An independent Student’s t-test was used to test for statistically significant differences in the following experiments: gene expression, protein expression, cell proliferation, colony formation, and tumorigenicity. A Chi-squared (*χ*^2^) test was used to calculate positive rate differences among normal liver tissue specimens, paracancerous liver tissue specimens, and HCC tumor specimens. A one-way analysis of variance (ANOVA) test was used to compare CCR10 transcript levels among normal liver tissue specimens, paracancerous liver tissue specimens, and HCC tumor specimens. We applied a *P*-value threshold of 0.05 for statistical significance to all statistical tests.

## Results

### CCR10 associated with inflammation-induced hepatocellular carcinogenesis

**Fig. 1:**
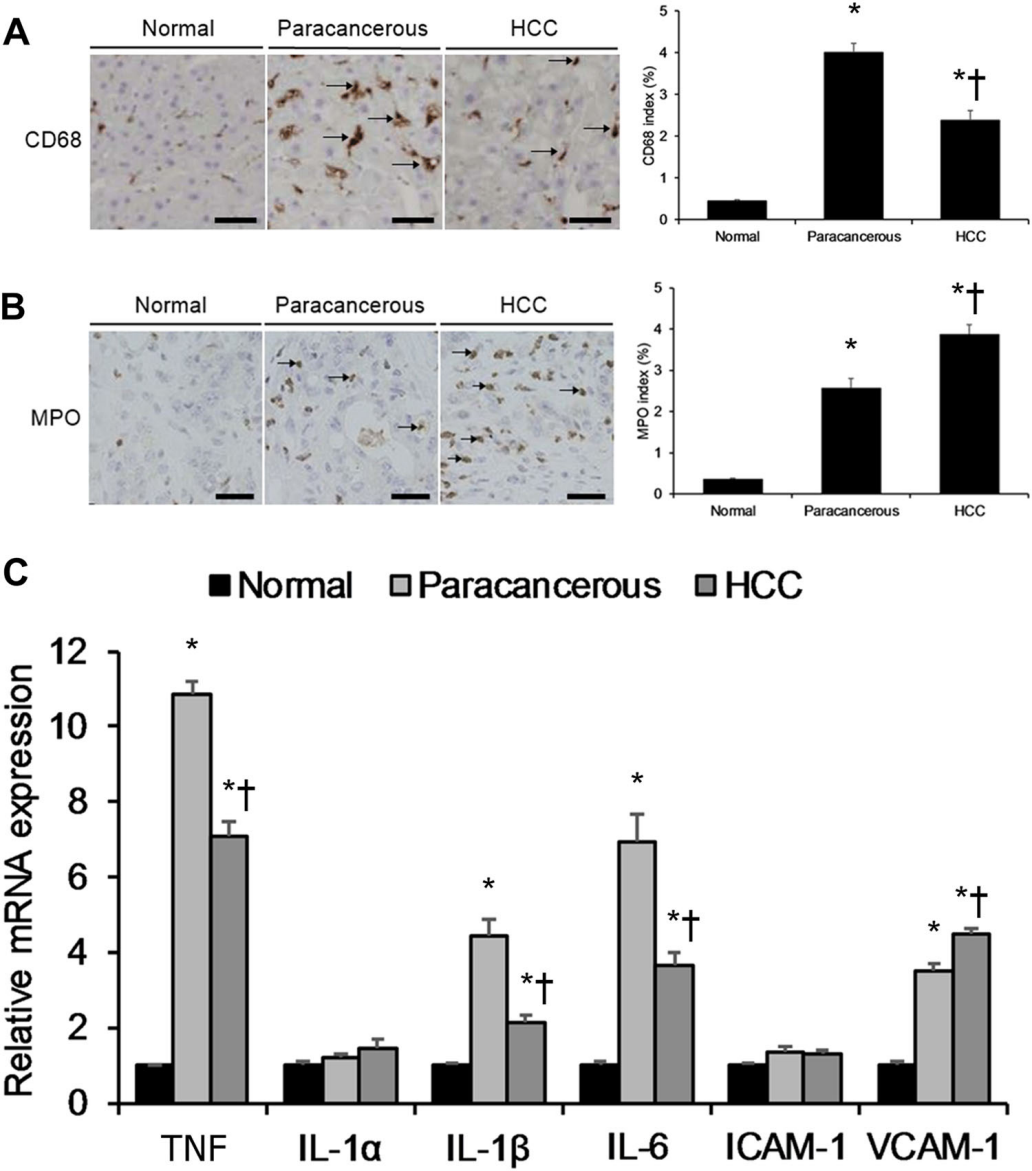
Human Hepatocarcinogenesis Associated with Inflammation. **a** Macrophagic CD68 staining of hepatocellular carcinoma (HCC) tumor specimens and matching paracancerous tissue specimens as well as normal liver specimens. The CD68 index (%) was calculated from counting CD68+ cells (brown-to-black) in 1000 cells per sample. Scale bar, 50 μm. **b** Neutrophilic MPO staining of HCC tumor specimens and matching paracancerous tissue specimens as well as normal liver specimens. The MPO index (%) was calculated from counting 1000 cells per sample. Scale bar, 50 μm. **c** Real-time RT-PCR analysis of key pro-inflammatory marker mRNA expression in HCC tumor specimens and matching paracancerous tissue specimens as well as normal liver specimens. *P < 0.05 vs. normal group, †*P* < 0.05 vs. paracancerous group. All values are reported as means ± standard deviations (SDs) for HCC tumor specimens and matching paracancerous tissue specimens (*n* = 10, randomly selected) as well as normal liver specimens (*n* = 10, randomly selected), respectively

Pro-inflammatory cytokines and inflammatory cells play important roles in HCC development^3^. In order to validate the association between hepatic carcinogenesis and inflammation, here we first re-performed several experiments previously performed by Yang et al.^6^. We assayed hepatic levels of CD68 (a marker of macrophagic infiltration) and myeloperoxidase (MPO) (a marker of neutrophil infiltration) in HCC tumor specimens, matching paracancerous specimens, and normal liver specimens. Both paracancerous specimens and HCC specimens displayed significantly higher levels of CD68 and MPO relative to normal liver specimens (Fig. 1a, b), demonstrating higher macrophagic and neutrophilic infiltration in paracancerous and HCC tissue. Moreover, both paracancerous and HCC specimens displayed significantly higher levels of pro-inflammatory cytokines (e.g., TNF, IL-1β, and IL-6) relative to normal liver specimens, with TNF (HCC: normal fold-change ratio = 7.09) showing the greatest fold-change (Fig. 1c).

**Fig. 2:**
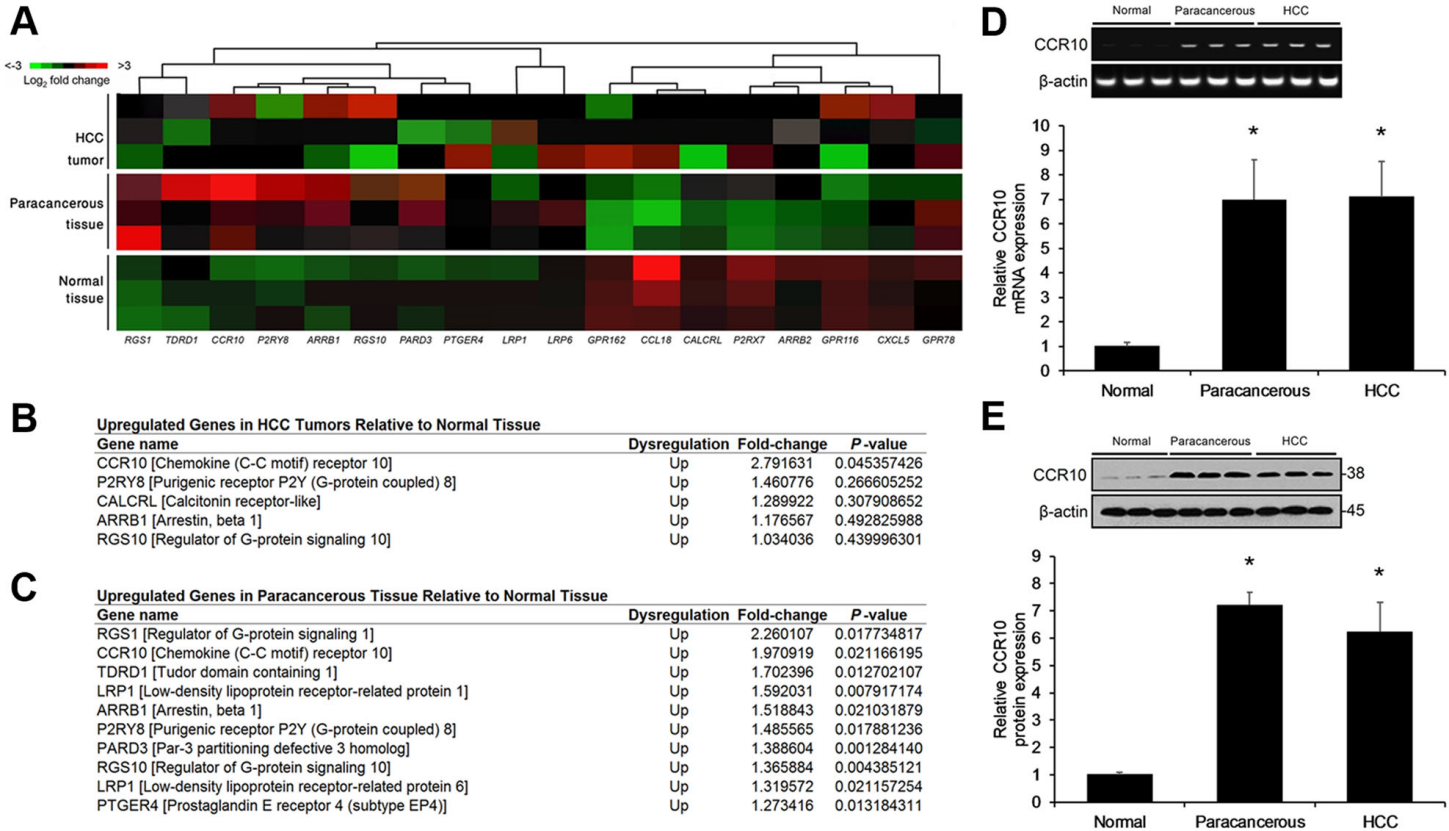
CCR10 Upregulation Associated with Inflammation-Driven Hepatocarcinogenesis. **a** Heatmap displaying the two-dimensional hierarchical clustering of hepatocellular carcinoma (HCC) tumor specimens and matching paracancerous tissue specimens (*n* = 3, randomly selected) as well as normal liver specimens (*n* = 3, randomly selected). As displayed in the color scale, fold-changes in mRNA expression relative to normal liver specimens are indicated in red (upregulation) and green (downregulation). **b** mRNA expression of upregulated GPCR-associated genes in HCC tumor specimens relative to normal liver specimens. **c** mRNA expression of upregulated GPCR-associated genes in paracancerous tissue specimens relative to normal liver specimens. **d** Real-time RT-PCR analysis of CCR10 mRNA expression in hepatocytes isolated from HCC tumor specimens and matching paracancerous tissue specimens as well as normal liver specimens. **e** Western blotting analysis of CCR10 protein expression in hepatocytes isolated from HCC tumor specimens and matching paracancerous tissue specimens as well as normal liver specimens.*P < 0.05 vs. normal group, †*P* < 0.05 vs. paracancerous group. All values are reported as means ± standard errors of the mean (SEMs) for HCC tumor specimens and matching paracancerous tissue specimens (*n* = 10, randomly selected) as well as normal liver specimens (*n* = 10, randomly selected), respectively

Various GPCRs have been shown to be dysregulated in HCC^5^. In order to validate the association between hepatic carcinogenesis and dysregulation in GPCR expression, here we re-performed a targeted gene expression microarray previously performed by Yang et al.^6^. Alterations in GPCR family gene expression were screened via a targeted gene expression microarray in three matching pairs of HCC and paracancerous specimens as well as three normal liver specimens. HCC and paracancerous specimens displayed significantly higher CCR10 expression relative to normal liver specimens (Fig. 2a–c). The fold-change of CCR10 expression in HCC vs. normal specimens (HCC: normal fold-change ratio = 2.79) was greater than the fold-change of CCR10 expression in paracancerous vs. normal specimens (paracancerous: normal fold-change ratio = 1.97) (Fig. 2a–c), suggesting that CCR10 may be associated with hepatocellular carcinogenesis.

CCR10 has been shown to be expressed on several cell types in the liver, most prominently infiltrating immune cells^18^. In order to remove the effects from hepatic infiltration of CCR10 + immune cells (or other CCR10 + cell types in the liver) and exclusively focus on the hepatocellular effects, we stringently isolated hepatocytes using a two-step collagenase isolation procedure followed by FACS (CD45−/CD95 + cell selection) for the remainder of this study^15,16^.

The isolated hepatocytes from human HCC and matching paracancerous specimens displayed significant CCR10 mRNA and protein upregulation relative to those isolated from normal liver specimens (Figs. 2d, e). Moreover, real-time RT-PCR of CD45 mRNA expression in the isolated hepatocyte populations revealed no discernable immune cell contamination (Supplementary Figure 2A, Online Supplementary Materials). Furthermore, FACS sorting by CCR10 expression revealed that normal isolated hepatocytes display negligible CCR10 expression, while isolated hepatocytes from paracancerous and HCC tissues display positive CCR10 expression (Supplementary Figure 3, Online Supplementary Materials). These findings suggest that hepatocellular CCR10 upregulation is associated with inflammation-induced hepatocellular carcinogenesis.

### Inflammation induces hepatocellular CCR10 expression

**Fig. 3:**
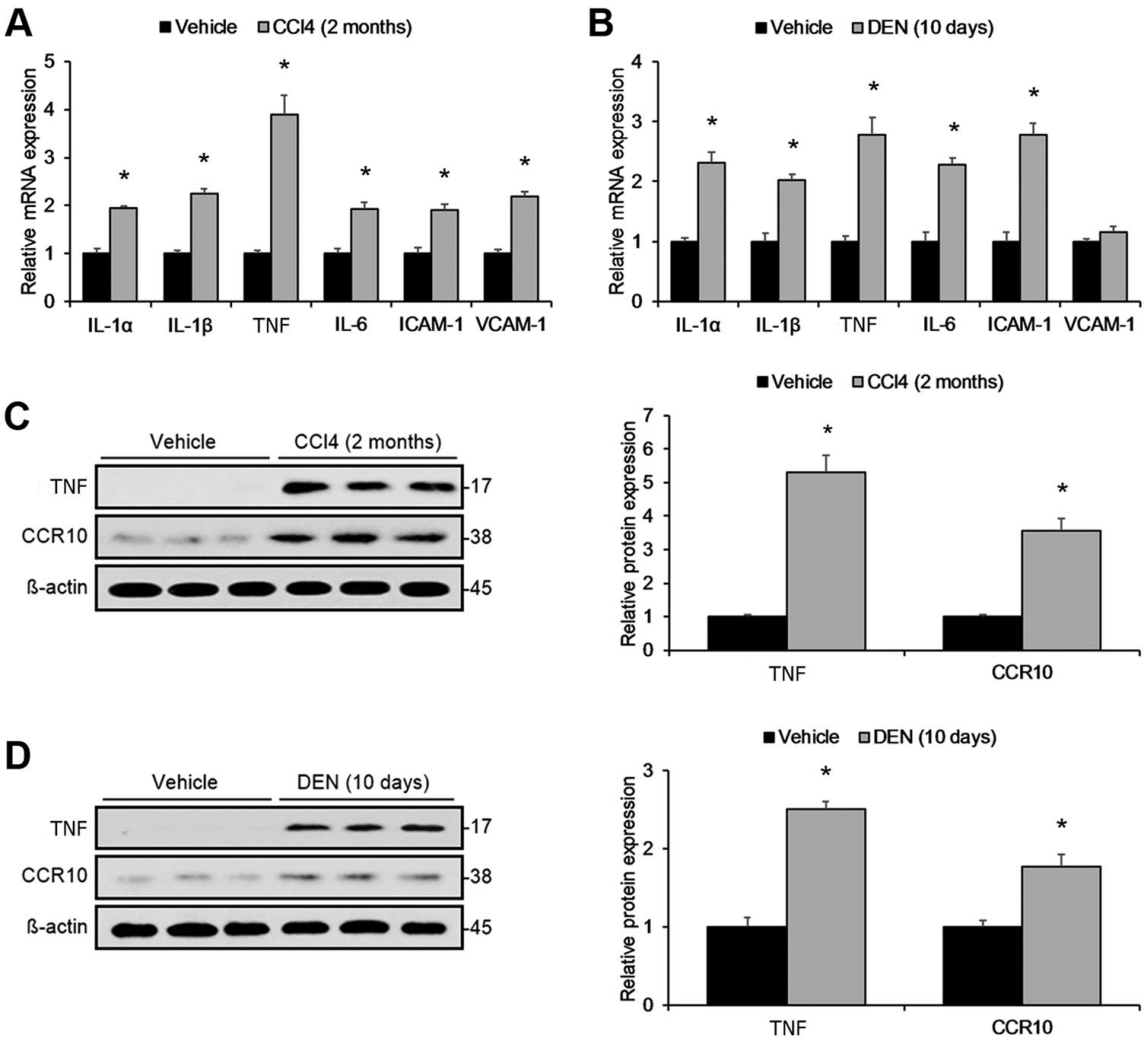
Inflammation-Driven CCR10 Upregulation in Wild-Type Murine Liver Tissue. **a** Real-time RT-PCR analysis of key pro-inflammatory marker mRNA expression in wild-type (WT) murine liver tissue after intraperitoneal (i.p.) injection regimen of either CCl_4_ or physiological saline (vehicle). **b** Real-time RT-PCR analysis of key pro-inflammatory marker mRNA expression in WT murine liver tissue after i.p. injection regimen of either DEN or physiological saline (vehicle). **c** Western blotting analysis of TNF and CCR10 protein expression in WT murine liver tissue after i.p. injection regimen of either CCl_4_ or physiological saline (vehicle). **d** Western blotting analysis of TNF and CCR10 protein expression in WT murine liver tissue after i.p. injection regimen of either DEN or physiological saline (vehicle). *P < 0.05 vs. vehicle group. All values are reported as means ± standard errors of the mean (SEMs). *n* = 12 mice in each group

Although CCR10 was found to be significantly upregulated in matching HCC and paracancerous specimens, it remained unclear as to whether CCR10 upregulation was induced via hepatic inflammation. Therefore, we constructed two murine models of hepatitis: one model was constructed through applying the hepatotoxin tetrachloromethane (CCl_4_), and the other model was constructed through applying the pro-inflammatory hepatocarcinogen diethylnitrosamine (DEN)^6^. The pro-inflammatory markers IL-1α, IL-1β, IL-6, TNF, ICAM-1, and VCAM-1 were all significantly upregulated under both hepatitis models (Fig. 3a, b). Both hepatitis models displayed significantly upregulated hepatocellular TNF and CCR10 protein expression (Fig. 3c, d). Real-time RT-PCR of CD45 mRNA expression in the isolated hepatocyte populations revealed no discernable immune cell contamination (Supplementary Figure 2B-C, Online Supplementary Materials). These findings reveal that hepatic inflammation induces hepatocellular CCR10 expression.

### TNF promotes CCR10 and CCL28 expression in a dose-dependent manner

**Fig. 4:**
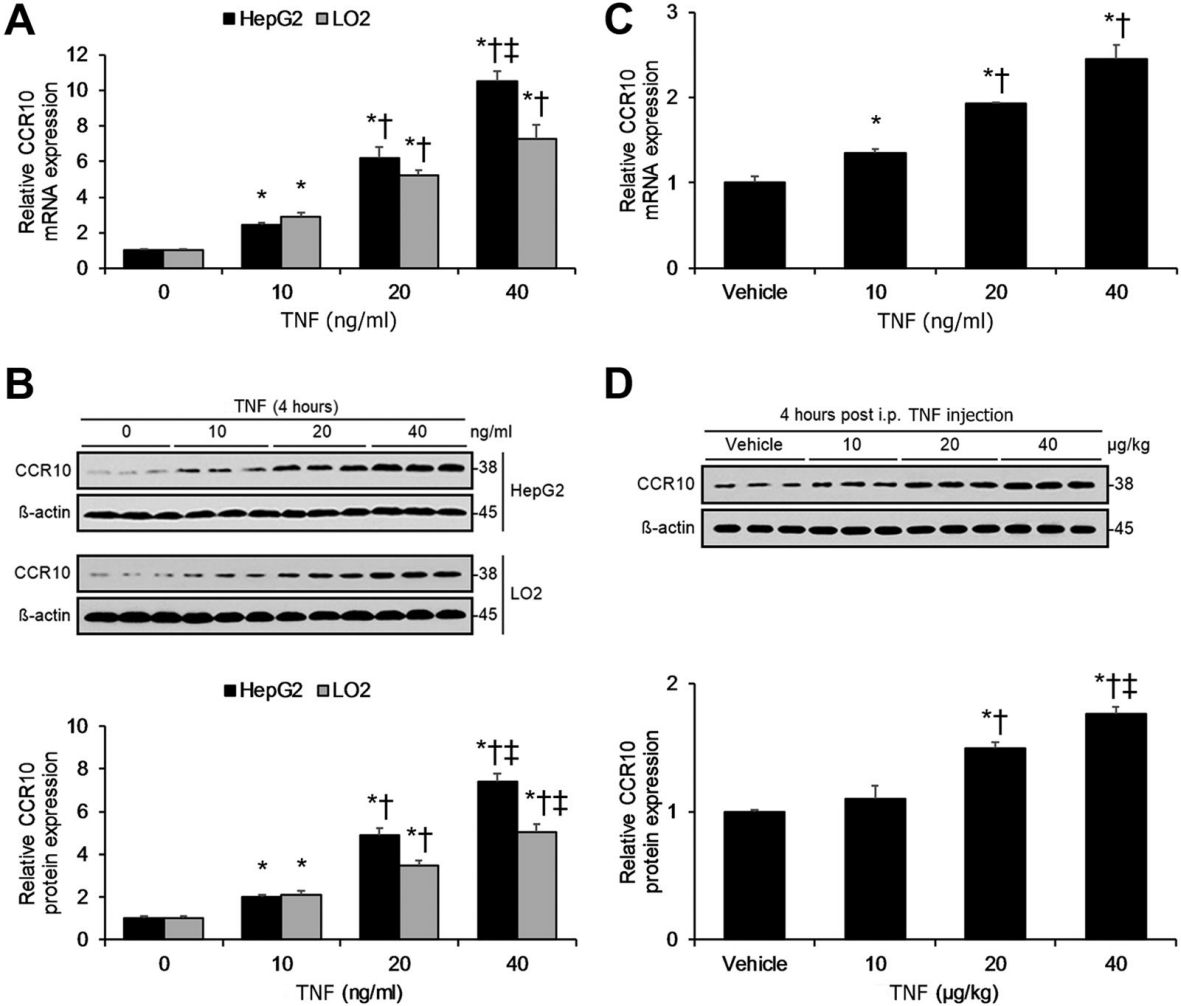
TNF Promotes Hepatocellular CCR10 Expression in a Dose-Dependent Manner. **a** Real-time RT-PCR analysis of CCR10 transcript expression and **b** Western blotting analysis of CCR10 protein expression in HepG2 and LO2 cells tissue 4 h after treatment with various doses of TNF. **c** Real-time RT-PCR analysis of CCR10 mRNA expression and **d** Western blotting analysis of CCR10 protein expression in isolated hepatocytes from WT murine liver tissue 4 h after i.p. injection with various doses of TNF. *P < 0.05 vs. 0 ng/ml (or µg/kg) group or vehicle group, †*P* < 0.05 vs. 10 ng/ml (or µg/kg) group, ‡*P* < 0.05 vs. 20 ng/ml (or µg/kg) group. All values are reported as means ± standard errors of the mean (SEMs). *n* = 12 mice in each group

The pro-inflammatory cytokine TNF has been closely associated with GPCR activity in hepatocytes^19^. As we have shown that inflammation induces hepatocellular CCR10 expression, we next investigated whether TNF alone promotes hepatocellular CCR10 expression. Application of TNF to the human HCC cell line HepG2 as well as the immortalized hepatocyte cell line LO2 significantly upregulated CCR10 expression in both cell lines in a dose-dependent manner (Fig. 4a, b). Moreover, injection of TNF into WT mice significantly raised isolated hepatocellular CCR10 expression in a dose-dependent manner (Fig. 4c, d).

As TNF promotes hepatocellular CCR10 expression in a dose-dependent manner, we next examined the gene expression and secretion of the two known natural ligand-agonists of CCR10, CCL27 and CCL28^20^. We found that HepG2 and LO2 cells both expressed and secreted CCL28, but not CCL27 (Supplementary Figure 4, Online Supplementary Materials). Additionally, application of TNF significantly upregulated CCL28 transcript expression and secretion in both cell lines in a dose-dependent manner (Supplementary Figure 4, Online Supplementary Materials).

### CCR10 contributes to hepatocellular carcinogenesis

**Fig. 5:**
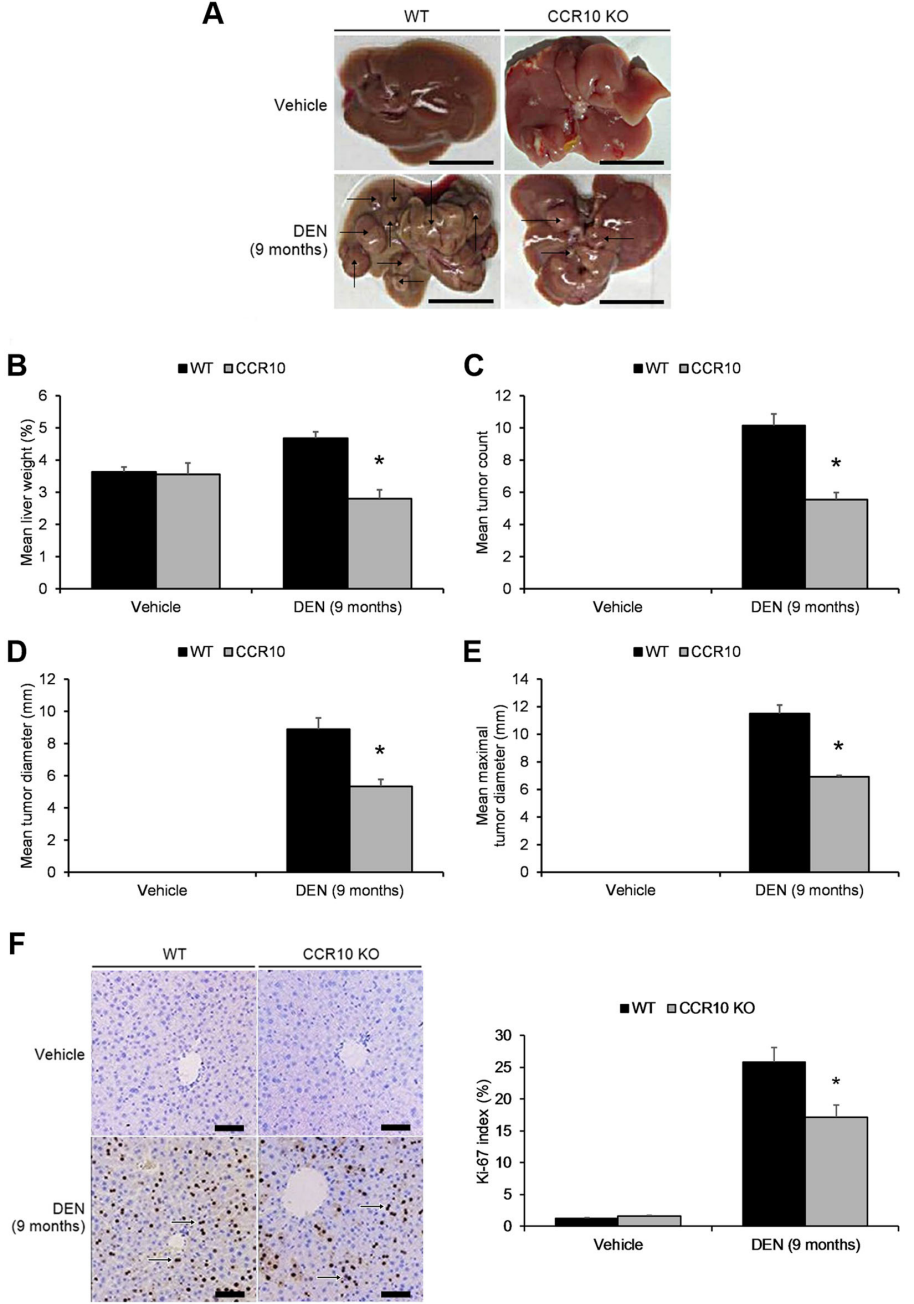
Knocking-Out CCR10 Suppresses DEN-Induced Hepatocarcinogenesis in Murine Livers. Livers were collected from CCR10 knockout (KO) and wild-type (WT) mice 9 months after intraperitoneal (i.p.) DEN injection. **a** Representative macroscopic images of DEN-induced HCC tumors extracted from vehicle-treated and DEN-treated CCR10 KO and WT mice. Scale bar, 10 mm. **b** Mean liver weight as a percentage of total body weight. **c** Mean HCC tumor count. **d** Mean diameter of HCC tumors. **e** Maximum diameter of HCC tumors. **f** The Ki-67 index (%) was calculated in DEN-induced HCC tumors by counting Ki-67 + hepatocytes (brown-to-black cells, as indicated by arrows) in 1000 cells per sample. Scale bar, 50 μm.*P < 0.05 vs. matching WT group. All values are reported as means ± standard errors of the mean (SEMs). *n* = 12 mice in each group

To validate whether CCR10 plays a contributory role in inflammation-induced hepatocellular carcinogenesis, a DEN-induced inflammatory hepatocarcinogenesis model was constructed in CCR10-knockout (CCR10 KO) mice and wild-type (WT) mice. Specifically, mice subjects were injected with DEN at 15 days of age; 9 months post-DEN injection, tumor sizes and tumor weights were measured. HCC tumors developed in all DEN-treated subjects (Fig. 5a). CCR10 KO mice showed a significantly lower liver weight/body weight ratio (Fig. 5b), significantly lower tumor incidence (Fig. 5c), and significantly lower mean and maximal tumor sizes relative to WT mice (Fig. 5d, e). In addition, Ki-67 immunohistochemical staining revealed that malignant hepatocellular proliferation was significantly reduced in CCR10 KO mice relative to WT mice (Fig. 5f). Moreover, CCR10 significantly raised xenograft tumor growth in Balb/c nude mice (Supplementary Figure 5, Online Supplementary Materials). These findings reveal that CCR10 contributes to DEN-induced inflammatory hepatocarcinogenesis.

### CCR10 promotes compensatory hepatocellular proliferation

**Fig. 6:**
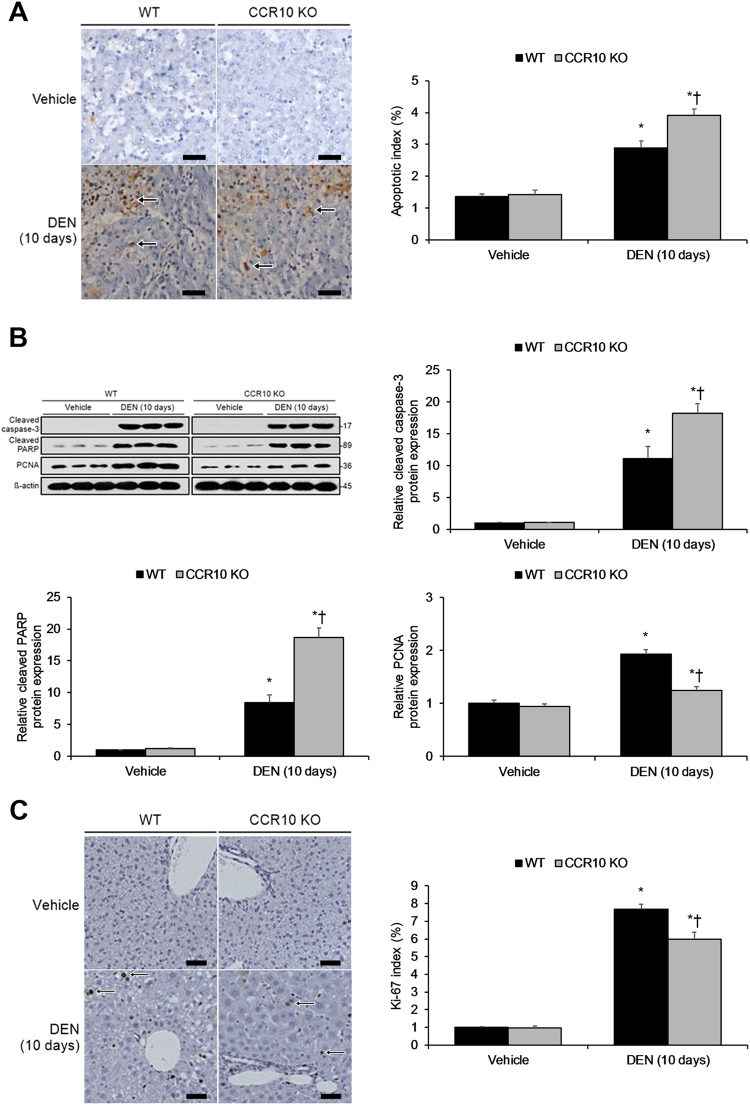
Knocking-Out CCR10 Promotes Inflammation-Driven Hepatocellular Apoptosis and Inhibits Hepatocellular Proliferation. Livers were collected from CCR10 knockout (KO) and wild-type (WT) mice 10 days after intraperitoneal (i.p.) injection of DEN or physiological saline (vehicle). **a** The apoptotic index (%) was calculated by counting TUNEL + cells (brown-to-black, as indicated by arrows) in 1000 cells per sample. Scale bar, 20 μm. **b** Western blotting analysis of cleaved caspase-3 protein expression, cleaved PARP protein expression, and PCNA protein expression. **c** The Ki-67 index was calculated by counting Ki-67 + cells (brown-to-black cells, as indicated by arrows) in 1000 cells per sample. Scale bar, 50 μm. *P < 0.05 vs. vehicle WT group, †*P* < 0.05 vs. DEN-treated WT group. All values are reported as means ± standard errors of the mean (SEMs). *n* = 12 mice in each group

Inflammation-driven hepatocarcinogenesis depends upon compensatory hepatocellular proliferation following hepatocellular apoptosis^21^. Thus, we investigated CCR10′s effects upon hepatocellular apoptosis as well as compensatory hepatocellular proliferation in the DEN-treated murine model of hepatitis. TUNEL staining revealed that DEN-treated mice displayed significantly higher apoptosis levels, with DEN-treated CCR10 KO mice showing significantly greater apoptosis levels (Fig. 6a). Accordingly, DEN-treated mice displayed significantly increased hepatocellular caspase-3 activation, PARP activation, and PCNA expression, with DEN-treated CCR10 KO mice showing significantly greater levels of all three markers (Fig. 6b). From Ki-67 staining, DEN-treated mice displayed significant upregulation of hepatocellular compensatory proliferation, with CCR10 KO mice showing significantly lower compensatory proliferation relative to WT mice (Fig. 6c). However, while DEN induced significantly enhanced hepatic MPO activity (a marker of neutrophilic infiltration), there was no significant difference in hepatic MPO activity between DEN-treated CCR10 KO and WT mice (Supplementary Figure 6, Online Supplementary Materials). These findings reveal that CCR10 reduces inflammation-driven hepatocellular apoptosis and promotes hepatocellular compensatory proliferation, but CCR10 does not significantly affect upstream neutrophilic infiltration.

### CCR10 drives in vitro hepatocellular proliferation through Akt phosphorylation

**Fig. 7:**
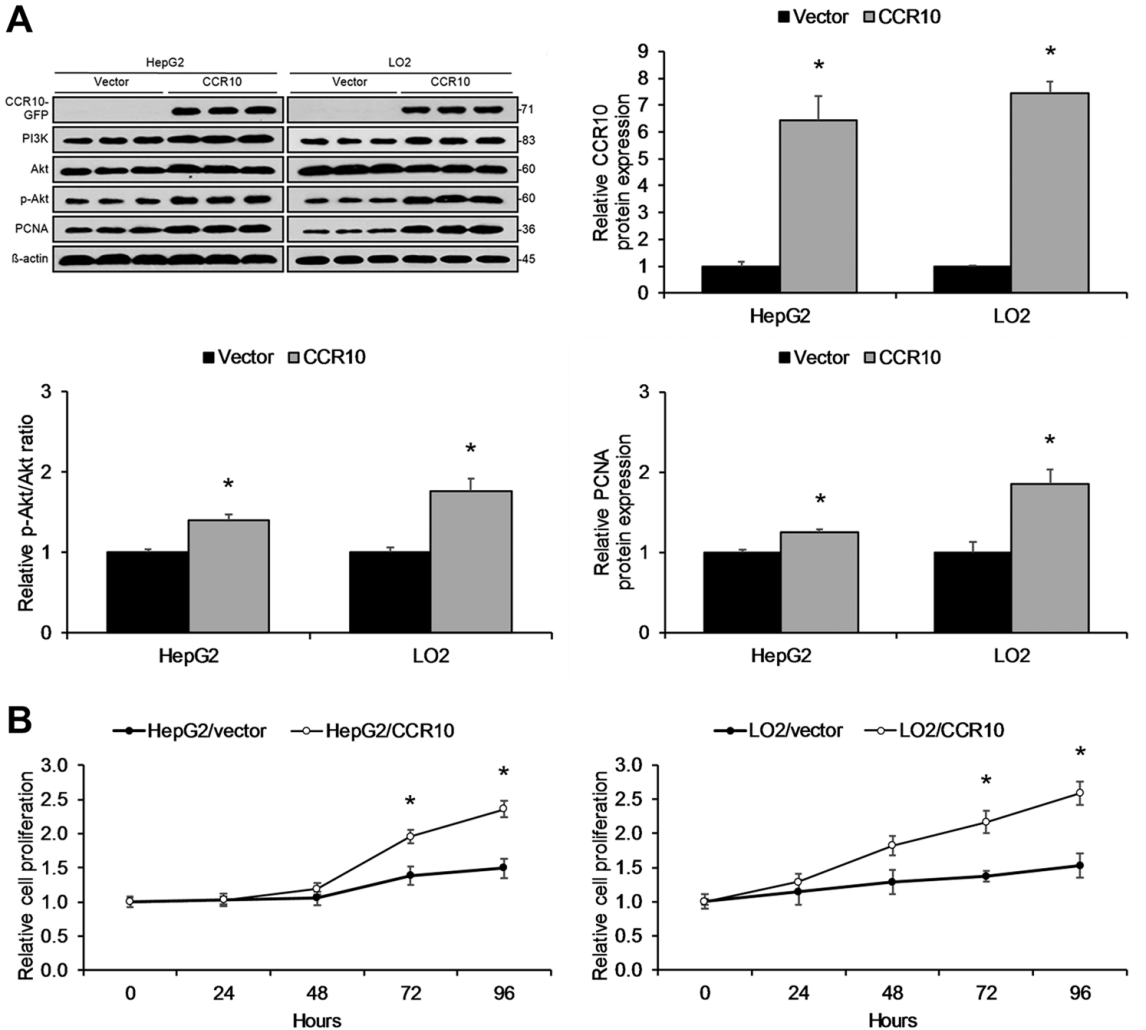
CCR10 Overexpression Promotes Hepatocellular Proliferation in Vitro. **a** CCR10 transfection into HepG2 and LO2 cells significantly increased Akt phosphorylation, PCNA protein expression, and **b** relative cell proliferation in both cell lines. Relative cell proliferation is defined as the fold-change in proliferation relative to the untreated parent cell line.*P < 0.05 vs. vector group. All values are reported as means ± standard errors of the mean (SEMs)

**Fig. 8:**
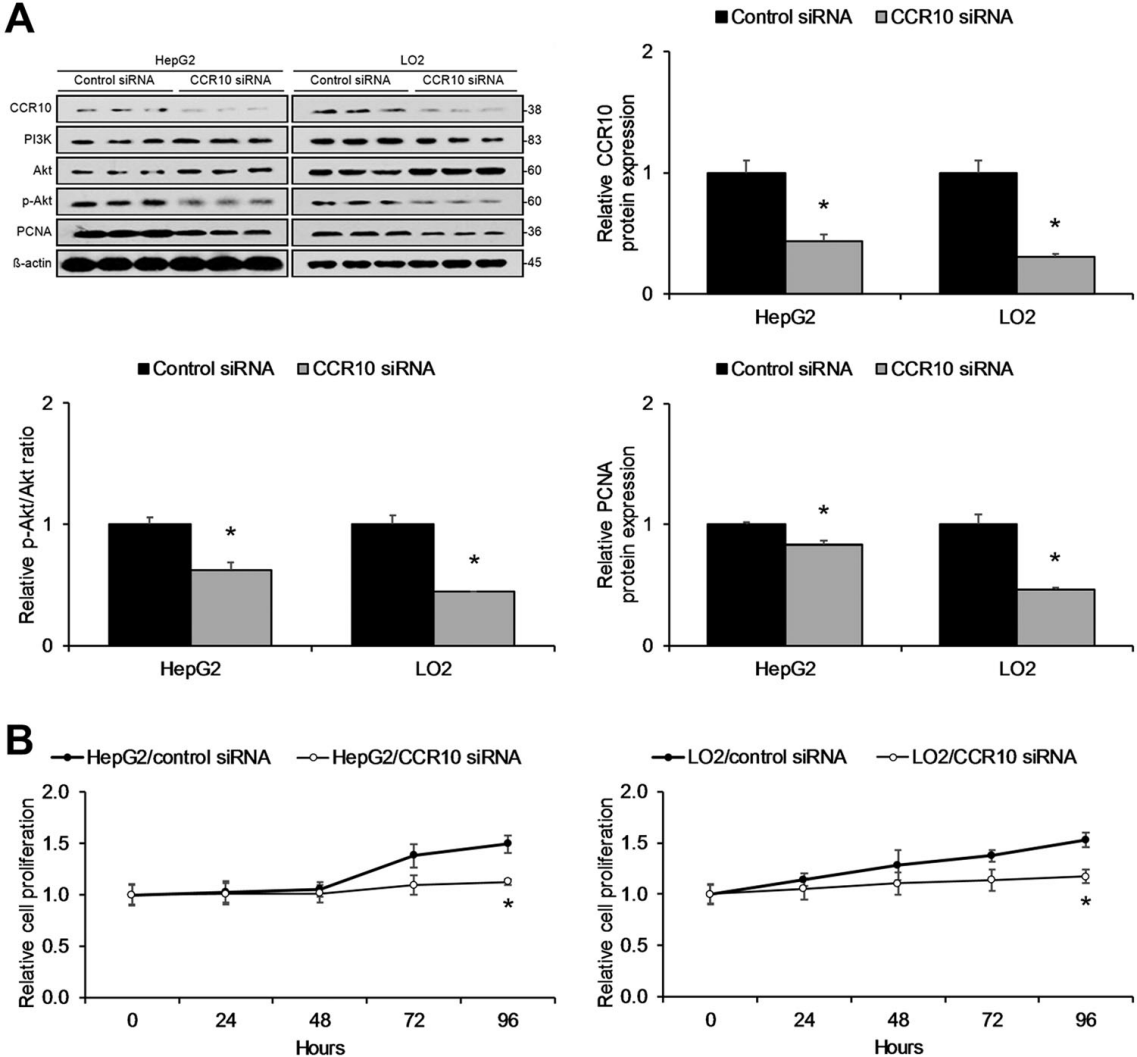
CCR10 Silencing Inhibits Hepatocellular Proliferation in Vitro. **a** CCR10 gene silencing by siRNA in HepG2 and LO2 cells significantly inhibited Akt phosphorylation, PCNA protein expression, and **b** relative cell proliferation in both cell lines. Relative cell proliferation is defined as the fold-change in proliferation relative to the untreated parent cell line. *P < 0.05 vs. vector group or control siRNA group. All values are reported as means ± standard errors of the mean (SEMs)

**Fig. 9:**
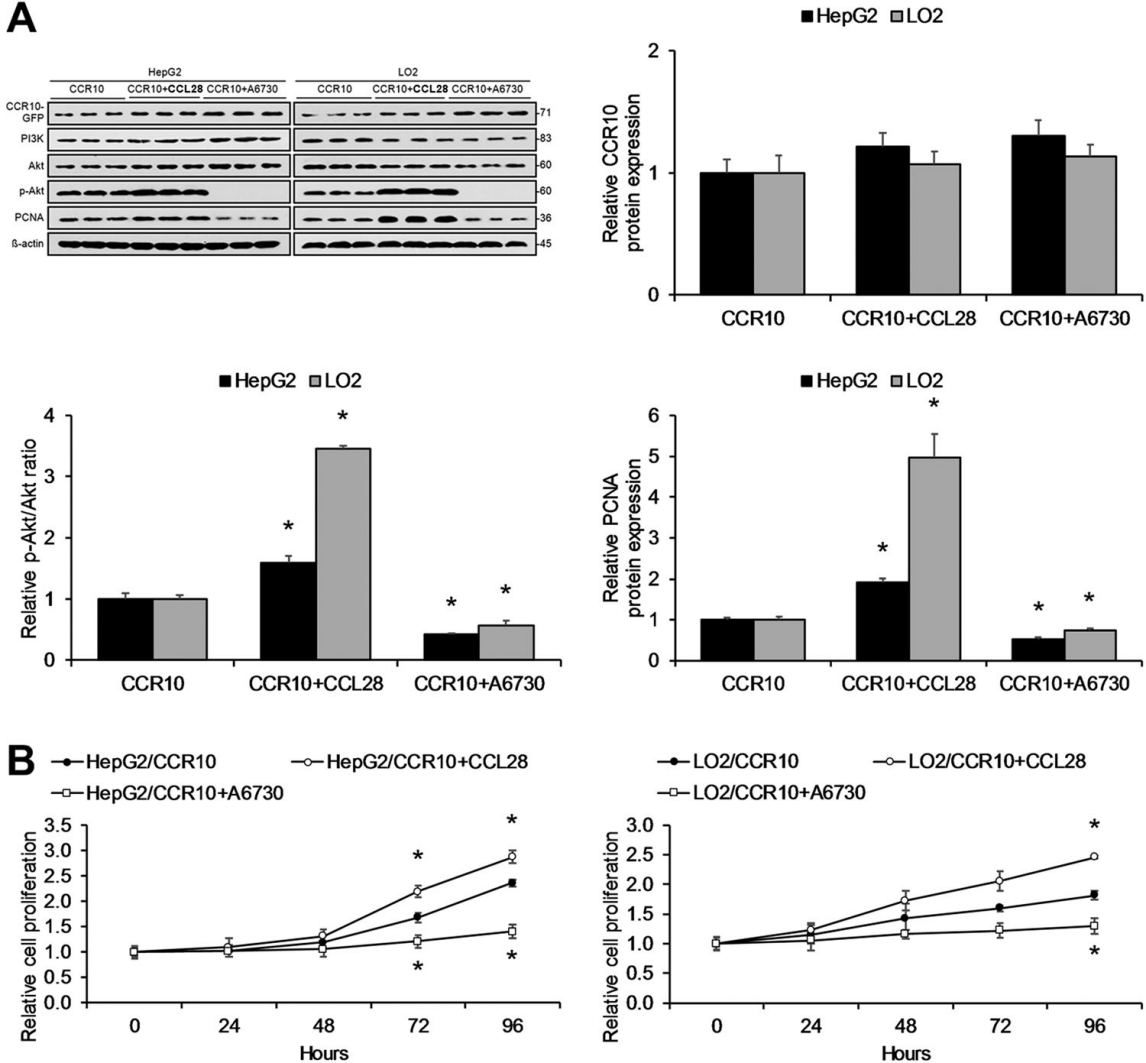
Activation of the CCL28-CCR10 Axis Promotes Hepatocellular Proliferation in Vitro. CCR10-transfected HepG2 and LO2 cells were treated with either the CCR10 agonist-ligand CCL28 or the Akt inhibitor A6730. **a** Activation of the CCL28-CCR10 axis by CCL28 significantly increased Akt phosphorylation, PCNA protein expression, and **b** relative cell proliferation in both cell lines, while Akt inhibition produced the opposite effects. Relative cell proliferation is defined as the fold-change in proliferation relative to the untreated parent cell line. *P < 0.05 vs. CCR10 group. All values are reported as means ± standard errors of the mean (SEMs)

HCC has been previously associated with the PI3K/Akt signaling pathway^22^, and CCR10 has been previously shown to drive PI3K/Akt pathway activation in response to ligand binding^17^.Thus, we investigated the effects of CCR10 upregulation on cell proliferation and PI3K/Akt pathway activation through transfection of a CCR10 plasmid into HepG2 and LO2 cell lines. CCR10 upregulation was found to significantly increase Akt phosphorylation, PCNA expression, and cell proliferation in both cell lines (Fig. 7). Next, we investigated the effects of CCR10 downregulation on cell proliferation and PI3K/Akt pathway activation through transducing a CCR10 siRNA into HepG2 and LO2 cell lines. CCR10 silencing significantly decreased Akt phosphorylation, PCNA expression, and cell proliferation in both cell lines (Fig. 8). Next, CCR10 plasmid-transfected HepG2 and LO2 cell lines were treated with the natural CCR10 ligand-agonist CCL28 or the Akt inhibitor A6730. CCR10 activation by CCL28 was found to significantly increase Akt phosphorylation, PCNA expression, and cell proliferation, while Akt inhibition by A6730 displayed the opposite effects (Fig. 9). These findings reveal that CCR10 drives in vitro hepatocellular proliferation via PI3K/Akt pathway activation.

### Short-term inflammation-driven hepatocellular proliferation dependent upon CCR10-mediated PI3K/Akt pathway activation

**Fig. 10:**
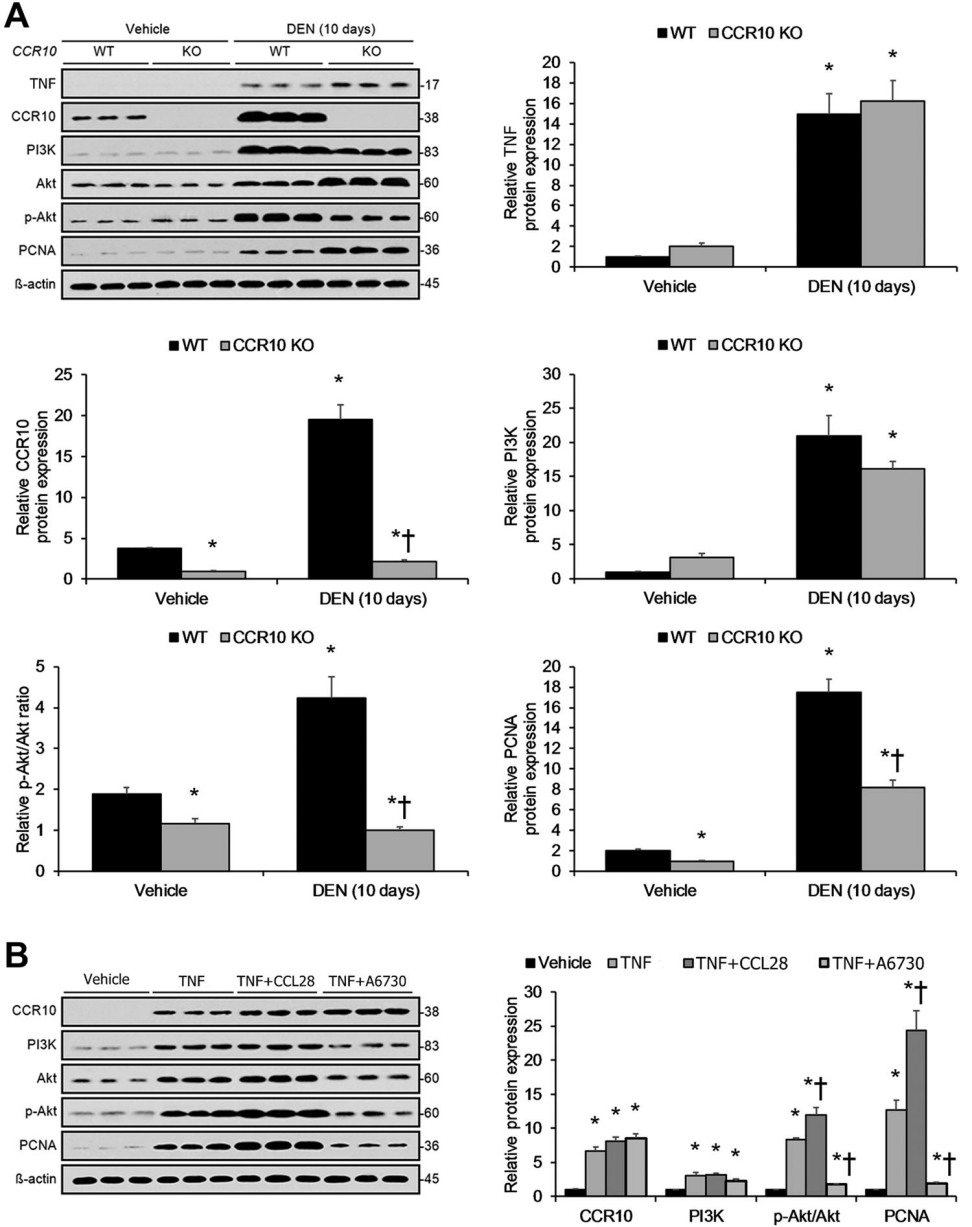
Short-Term Inflammation-Driven Hepatocellular Proliferation Dependent upon CCR10-Mediated PI3K/Akt Pathway Activation. Following short-term DEN-induced inflammation (10 days after i.p. DEN injection), (**a**) Western blotting analysis showed significantly enhanced TNF protein expression, CCR10 protein expression, PI3K protein expression, Akt phosphorylation, and PCNA protein expression. Knocking-out CCR10 significantly opposed these inflammation-induced effects but did not significantly affect TNF or PI3K protein expression.*P < 0.05 vs. vehicle WT group, †*P* < 0.05 vs. DEN-treated WT group. **b** Western blotting analysis of CCR10 protein expression, PI3K protein expression, Akt phosphorylation, and PCNA protein expression in murine liver tissue 6 h after intraperitoneal (i.p.) injection of TNF, which produced significant increases in CCR10 protein expression, PI3K protein expression, Akt phosphorylation, and PCNA protein expression. Pretreatment with the CCR10 agonist-ligand CCL28 significantly increased Akt phosphorylation and PCNA expression levels, while pretreatment with the Akt inhibitor A6730 produced the opposite effects. Neither CCL28 nor A6730 had any significant effect upon CCR10 or PI3K expression. *P < 0.05 vs. vehicle group, †*P* < 0.05 vs. TNF group. All values are reported as means ± standard errors of the mean (SEMs). *n* = 12 mice in each group

To understand CCR10′s role in hepatocellular proliferation and PI3K/Akt pathway activation in reaction to short-term DEN-induced inflammation in vivo, we investigated hepatocellular PI3K/Akt pathway activity in short-term DEN-treated CCR10 KO and WT mice. Following short-term DEN-induced hepatic inflammation (10 days), hepatocytes isolated from CCR10 KO mice showed significantly lower DEN-induced Akt phosphorylation and PCNA expression relative to hepatocytes isolated WT mice (Fig. 10a). CCR10 KO did not significantly perturb TNF or PI3K expression (Fig. 10a).

To validate the above findings, we next investigated hepatocellular PI3K/Akt pathway activity in reaction to short-term TNF exposure. Injection of TNF into WT mice significantly elevated CCR10 expression as well as Akt phosphorylation in isolated hepatocytes (Fig. 10b). Notably, pretreating mice with the natural CCR10 ligand-agonist CCL28 significantly increased hepatocellular Akt phosphorylation and PCNA expression, while pretreatment with the Akt inhibitor A6730 had the opposite effects (Fig. 10b). These findings reveal that short-term inflammation-induced malignant hepatocellular proliferation is dependent upon on CCR10-driven PI3K/Akt pathway activation.

### Long-term inflammation-induced HCC dependent upon CCR10/PI3K/Akt pathway activation

**Fig. 11:**
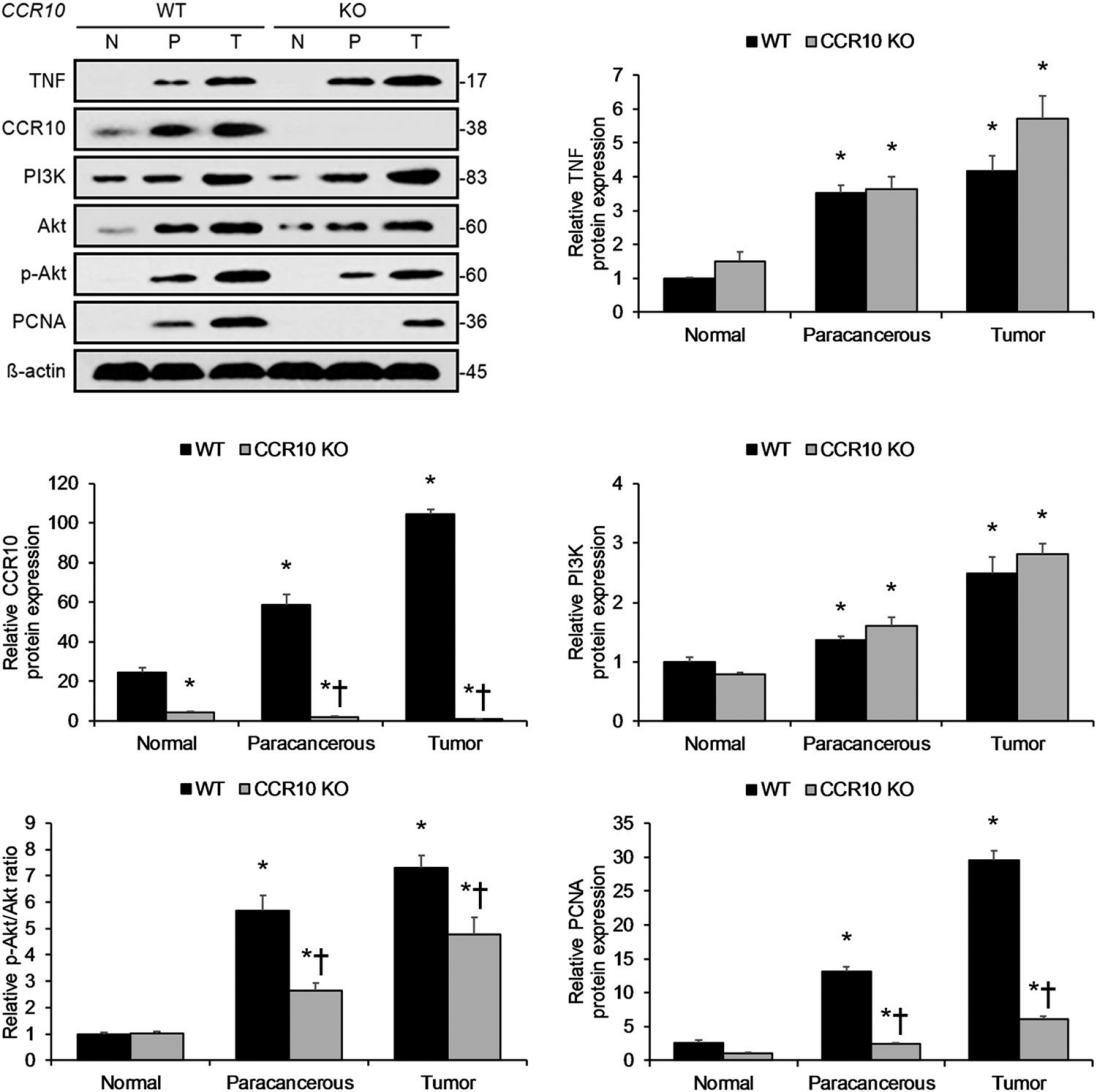
Long-Term Inflammation-Driven Hepatocarcinogenesis Dependent upon CCR10-Mediated PI3K/Akt Pathway Activation. Following long-term DEN-induced inflammatory hepatocarcinogenesis (9 months after i.p. DEN injection), Western blotting analysis showed HCC tumors and matching paracancerous tissue displayed significantly enhanced TNF protein expression, CCR10 protein expression, PI3K protein expression, Akt phosphorylation, and PCNA protein expression over normal liver tissue. Knocking-out CCR10 significantly opposed these inflammation-induced effects but did not significantly affect TNF or PI3K protein expression. *P < 0.05 vs. normal WT group, †*P* < 0.05 vs. matching WT group. All values are reported as means ± standard errors of the mean (SEMs). *n* = 12 mice in each group

To understand CCR10′s role in hepatocellular proliferation and PI3K/Akt pathway activation in reaction to long-term DEN-induced inflammatory hepatocarcinogenesis, we next investigated hepatocellular PI3K/Akt pathway activity in long-term DEN-treated CCR10 KO and WT mice. Following long-term DEN-induced inflammatory hepatocarcinogenesis (9 months), hepatocytes isolated from HCC tumor tissue or surrounding paracancerous tissue displayed significant upregulation in CCR10 expression, PI3K expression, Akt expression, Akt phosphorylation, and PCNA expression relative to hepatocytes isolated from matching normal liver tissue (Fig. 11). Hepatocytes isolated from HCC tumor tissue and matching paracancerous tissue in CCR10 KO mice showed significantly lower Akt phosphorylation and PCNA expression relative to hepatocytes isolated from their WT counterparts (Fig. 11). Knocking-out CCR10 did not significantly perturb PI3K or Akt expression (Fig. 11). These findings reveal that long-term inflammation-induced hepatocarcinogenesis is dependent upon on CCR10-driven PI3K/Akt pathway activation.

## Discussion

Improving our understanding of key pathways involved in inflammation-driven hepatocarcinogenesis remains an important goal of current HCC research^23,24^. In this study, we demonstrated that hepatic inflammation drives production of the pro-inflammatory cytokine TNF, which promotes hepatocellular CCR10 expression. In turn, we also showed that hepatocellular CCR10 activates PI3K/Akt pathway signaling via phosphorylation of Akt, which drives hepatocarcinogenesis through inhibiting apoptosis and promoting compensatory proliferation. Our findings reveal that inflammation-driven hepatocarcinogenesis operates via activation of the CCR10/PI3K/Akt axis and that CCR10 is a key promoter of inflammation-driven hepatocarcinogenesis.

Inflammatory processes play a critical role in HCC development^23,24^. TNF has been established as a key promoter of inflammation-driven hepatocarcinogenesis in NAFLD, alcoholic cirrhosis, and chronic viral hepatitis^25–27^. Although liver inflammation has been shown to drive hepatocarcinogenesis via TNF in DEN-induced and CCl_4_-induced murine models of hepatitis, TNF’s downstream target(s) that promote tumorigenic change in hepatocytes remain largely unknown. However, it is known that TNF-driven liver inflammation is associated with dysregulated expression of GPCR-associated proteins. Here, we initially observed elevated levels of pro-inflammatory cytokines (most notably TNF), infiltration of inflammatory cells, and upregulation of four GPCR-associated genes (CCR10, P2RY8, ARRB1, and RGS10) in human HCC tumor and paracancerous specimens. Notably, all four GPCR-associated genes have been previously associated with human carcinogenesis^,28–30^. Through RT-PCR and Western blotting validation, we confirmed that CCR10 expression was significantly elevated in hepatocytes isolated from matching human HCC tumor and paracancerous specimens. Moreover, FACS revealed strong CCR10 expression in paracancerous and HCC hepatocytes with negligible CCR10 expression in normal hepatocytes.

Although these initial findings suggested an association between CCR10 upregulation and inflammation-driven hepatocarcinogenesis, it remained unclear as to whether CCR10 upregulation was induced via hepatic inflammation. Employing two well-established models of murine hepatitis (DEN and CCl_4_)^6^, we next demonstrated that DEN-induced and CCl_4_-induced hepatitis significantly elevated hepatocellular TNF and CCR10 expression in WT mice. In addition, TNF significantly promoted the expression of hepatocellular CCR10 and its natural ligand-agonist CCL28 in a dose-dependent manner. Accordingly, TNF has been shown to induce pro-inflammatory chemokine and cytokine expression by HCC progenitor cells and surrounding cells that directly contributes to HCC oncogenesis^31^. These combined findings suggest that hepatic inflammation drives hepatocellular TNF and CCR10 upregulation and that CCR10 upregulation lies downstream of TNF.

Having established that CCR10 is significantly upregulated in short-term murine models of hepatitis, we next investigated CCR10′s role in long-term DEN-induced murine hepatocarcinogenesis using CCR10 KO and WT mice. Nine months after DEN treatment, tumor counts and sizes were both significantly reduced in CCR10 KO mice relative to WT mice. Moreover, implantation of CCR10-transfected Hep3B xenograft tumors in nude mice significantly promoted in vivo tumor growth. Similarly, previous work has shown that activation of CCR10 contributes to carcinogenesis and invasive progression in melanoma and glioma cells in vivo^17,32^. These findings suggest that CCR10 is a key contributor to inflammation-driven hepatocarcinogenesis.

Previous research suggests that hepatocarcinogenesis is buttressed by compensatory proliferation of hepatocytes following apoptosis^33^. Thus, we next investigated CCR10′s effects upon hepatocellular apoptosis as well as compensatory hepatocellular proliferation in DEN-treated CCR10 KO and WT mice. Knocking-out CCR10 produces significantly greater hepatocellular apoptosis levels along with significantly lower compensatory proliferation in vivo. Consistent with our present findings, CCR10 activation has been associated with promoting the development of malignant melanoma and glioma through stimulating cell proliferation^17,32^.

Moreover, previous studies in malignant melanoma and glioma suggest that CCR10 promotes cellular proliferation through PI3K/Akt pathway activation^17,32^. With respect to HCC, Akt phosphorylation has also been shown to be a key factor in hepatocarcinogenesis, with p-Akt levels positively correlating with inferior outcomes in HCC patients^34^. Accordingly, here we found that artificial CCR10 expression significantly enhanced hepatocellular Akt phosphorylation and expression of the cell proliferation marker PCNA in vitro. Moreover, employing CCR10′s natural ligand-agonist CCL28^35^ that we demonstrated to be expressed and secreted by hepatocytes in vitro, we found that application of CCL28 significantly promoted CCR10-driven hepatocellular Akt phosphorylation and PCNA expression in vitro, while application of the Akt inhibitor A6730 produced the opposite effects. Validating these in vitro findings, we confirmed that short-term DEN-treated CCR10 KO mice displayed significantly lower levels of hepatocellular Akt phosphorylation and PCNA expression. Notably, however, DEN-treated CCR10 KO and WT mice showed similar levels of hepatocellular TNF expression. Moreover, short-term stimulation with TNF significantly enhanced hepatocellular CCR10 expression, Akt phosphorylation, and PCNA expression, supporting our claim that CCR10/PI3K/Akt activation lies downstream of TNF. In order to investigate the longer-term effects of CCR10 expression, we finally investigated hepatocellular PI3K/Akt pathway activity in long-term DEN-treated CCR10 KO and WT mice. Consistent with our short-term findings, HCC tumor tissue and matching paracancerous tissue in long-term DEN-treated CCR10 KO mice displayed significantly lowered hepatocellular Akt phosphorylation and PCNA expression. These combined findings indicate that CCR10/PI3K/Akt pathway activation occurs at the pre-malignant/malignant hepatocyte level. That being said, this evidence does not completely rule out ancillary upstream effects from leukocytes or other cell types that may also contribute to HCC development.

In sum, these findings demonstrate that hepatic inflammation drives production of the pro-inflammatory cytokine TNF, which promotes hepatocellular CCR10 expression and downstream PI3K/Akt-mediated hepatocarcinogenesis. These findings reveal that inflammation-driven hepatocarcinogenesis operates via activation of the CCR10/PI3K/Akt axis. As CCR10 appears to function as a linkage between TNF stimulation and downstream PI3K/Akt pathway activation, CCR10 may show promise as a potential therapeutic target for inflammation-driven HCC.

### Data availability

All data generated or analyzed during this study are included in this published article and its supplementary information files.

## Electronic supplementary material

Supplementary Figure 1

Supplementary Figure 2

Supplementary Figure 3

Supplementary Figure 4

Supplementary Figure 5

Supplementary Figure 6

Supplementary Information
