# Supplementary figures and images for: The chemokine receptor CCR10 promotes inflammation-driven hepatocarcinogenesis via PI3K/Akt pathway activation
Source: Cell Death Dis. 2018 Feb 14;9(2):232. doi: 10.1038/s41419-018-0267-9 (PMC5833857; doi:10.1038/s41419-018-0267-9)

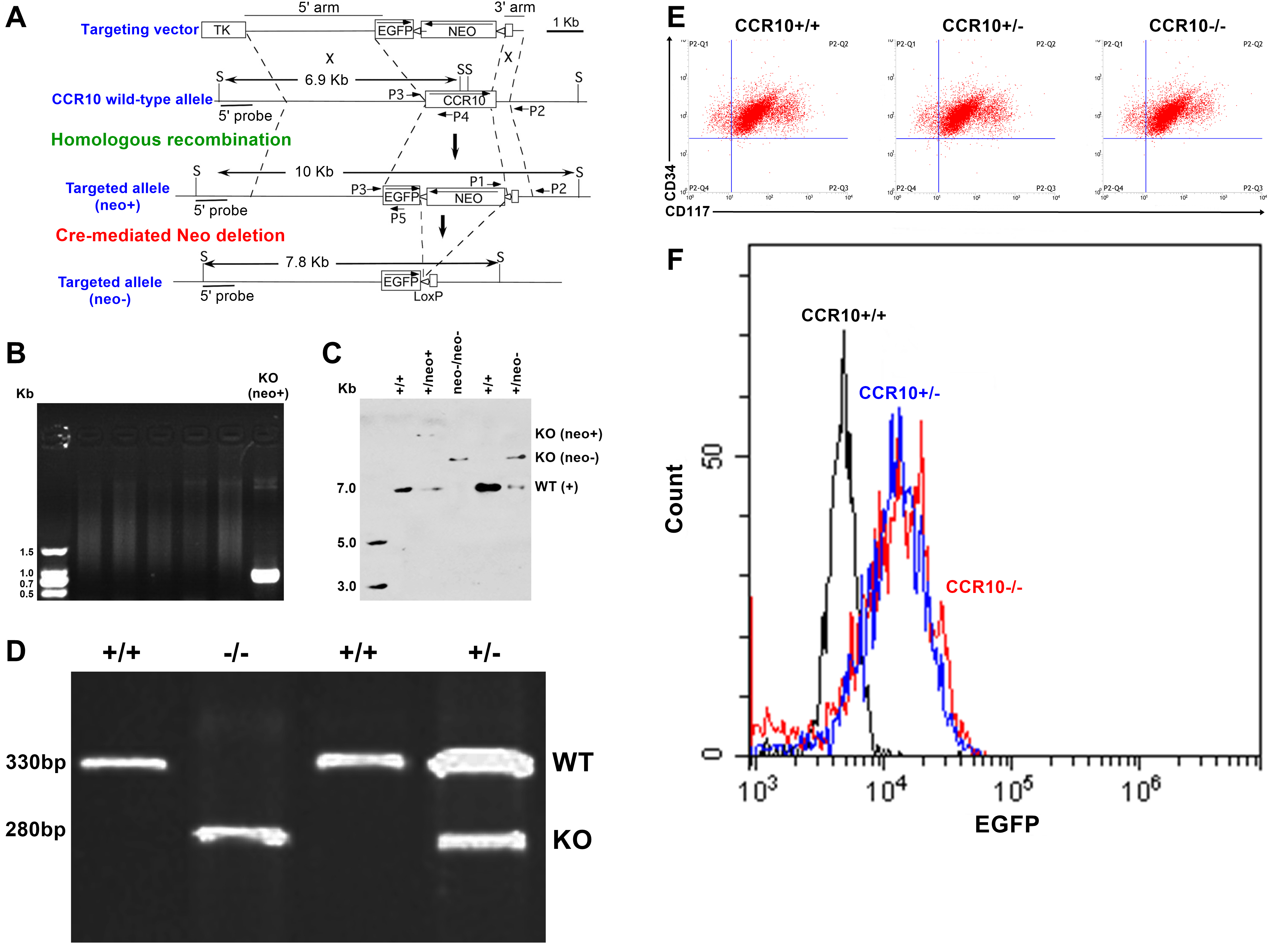

Supplement: Supplementary file 1 — Supplementary Figure 1 [file 41419_2018_267_MOESM1_ESM.tif]

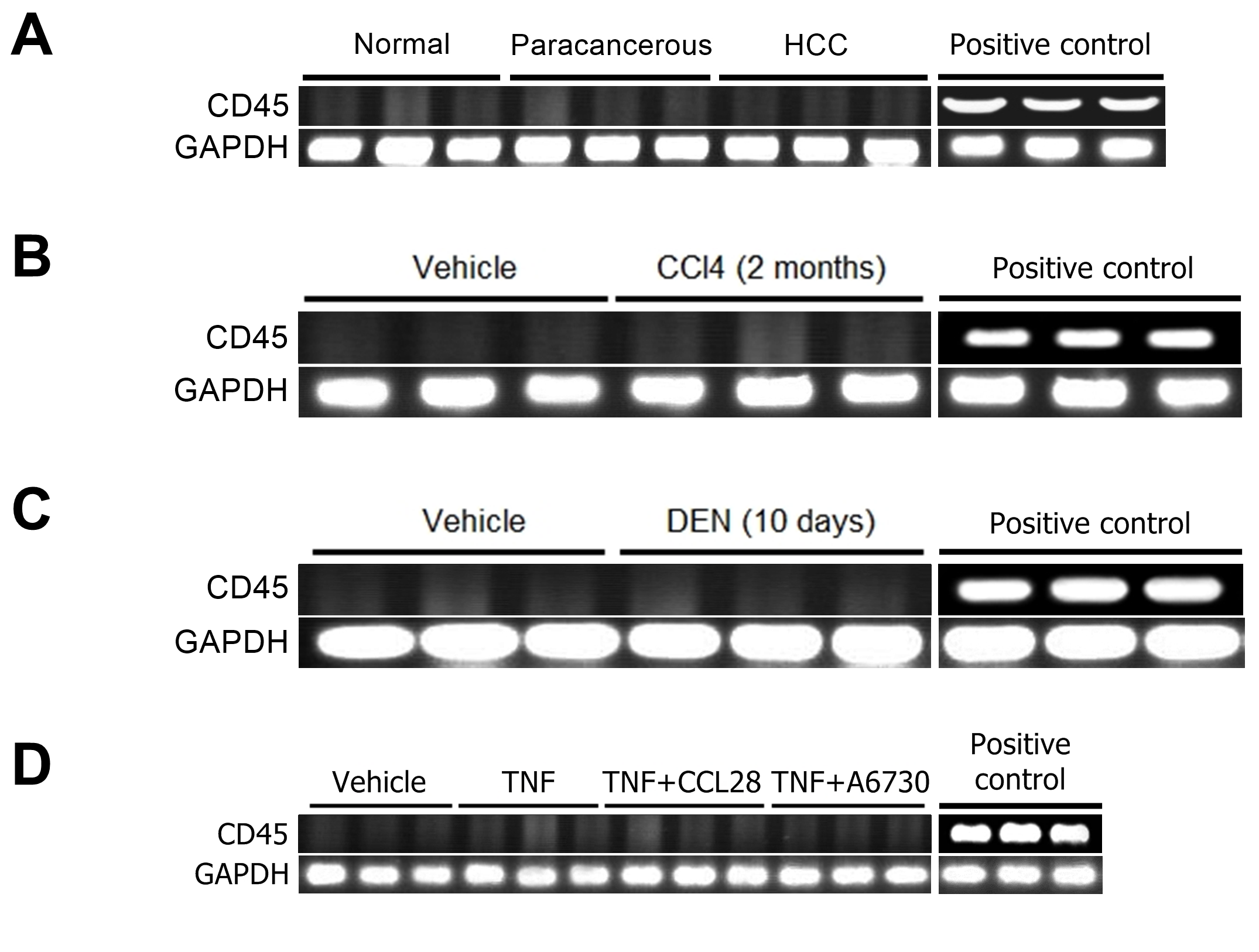

Supplement: Supplementary file 2 — Supplementary Figure 2 [file 41419_2018_267_MOESM2_ESM.tif]

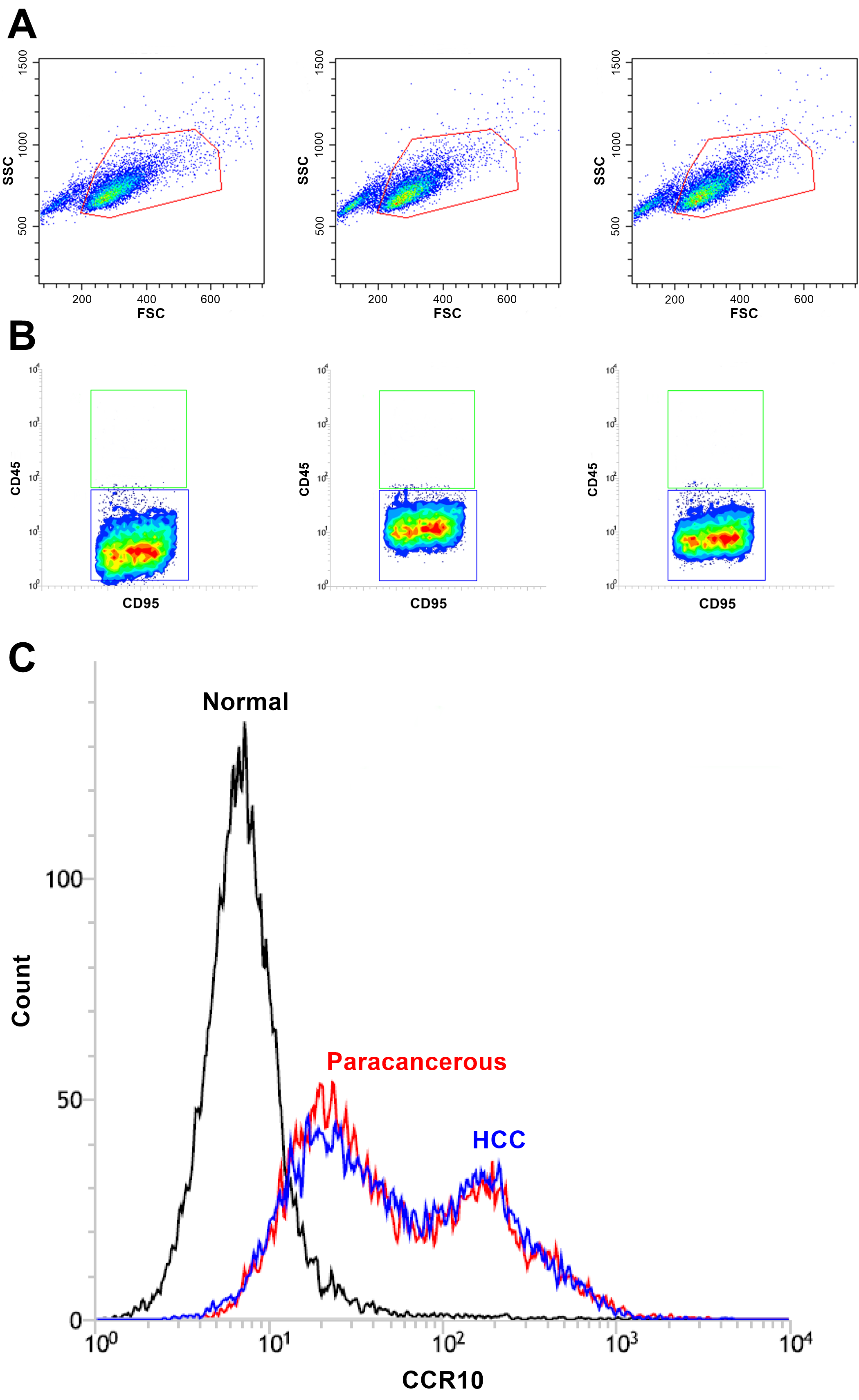

Supplement: Supplementary file 3 — Supplementary Figure 3 [file 41419_2018_267_MOESM3_ESM.tif]

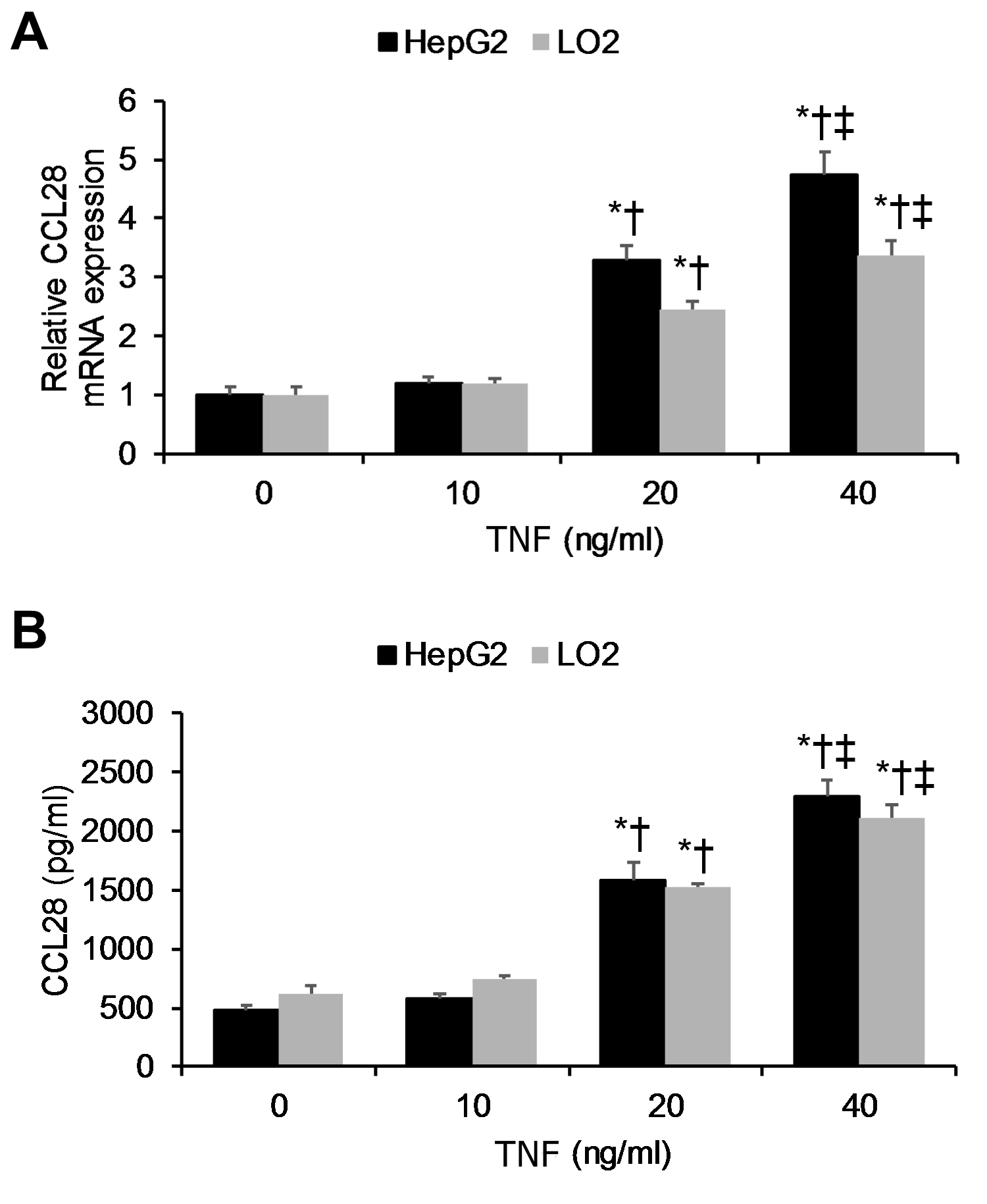

Supplement: Supplementary file 4 — Supplementary Figure 4 [file 41419_2018_267_MOESM4_ESM.tif]

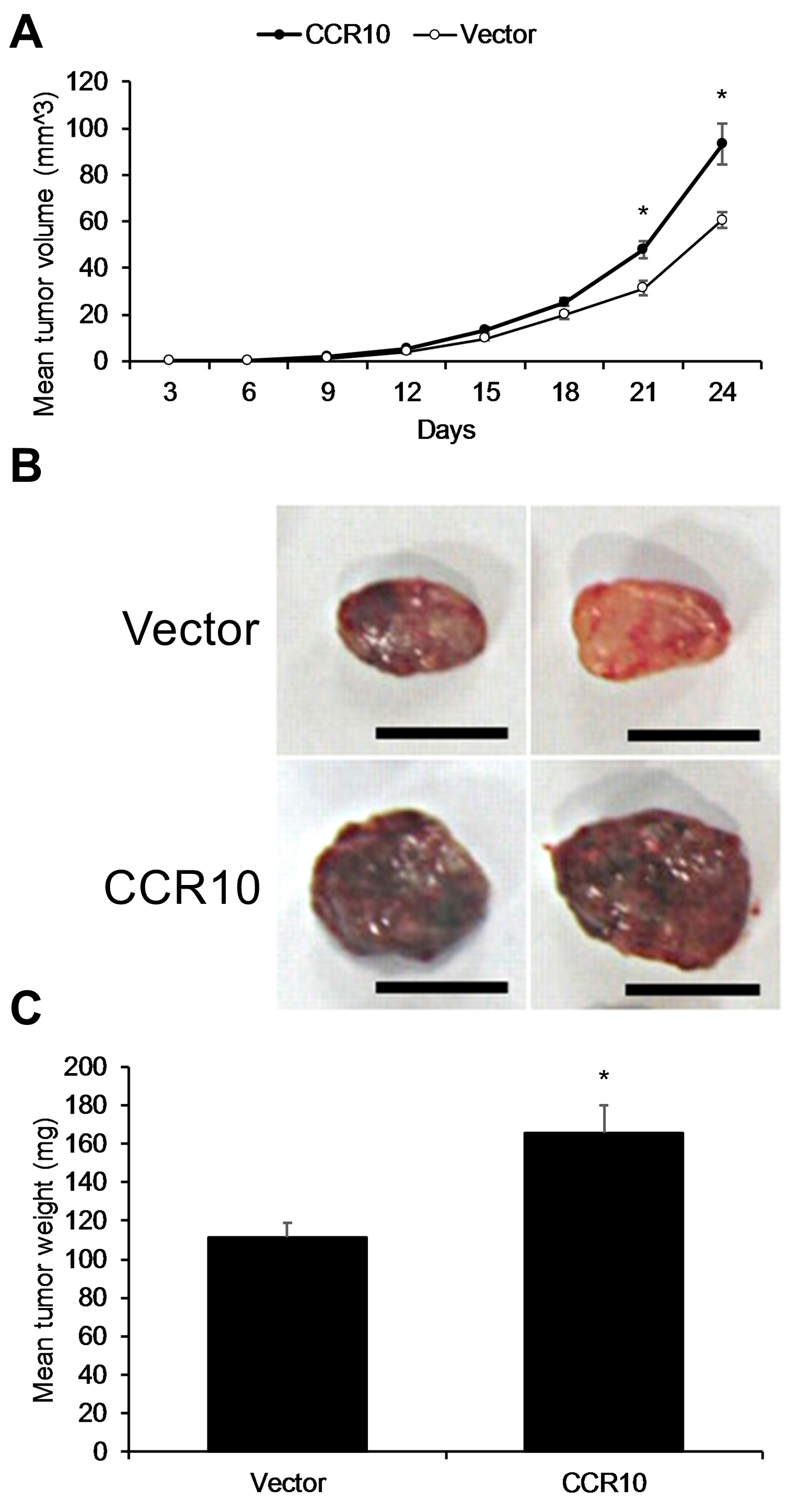

Supplement: Supplementary file 5 — Supplementary Figure 5 [file 41419_2018_267_MOESM5_ESM.tif]

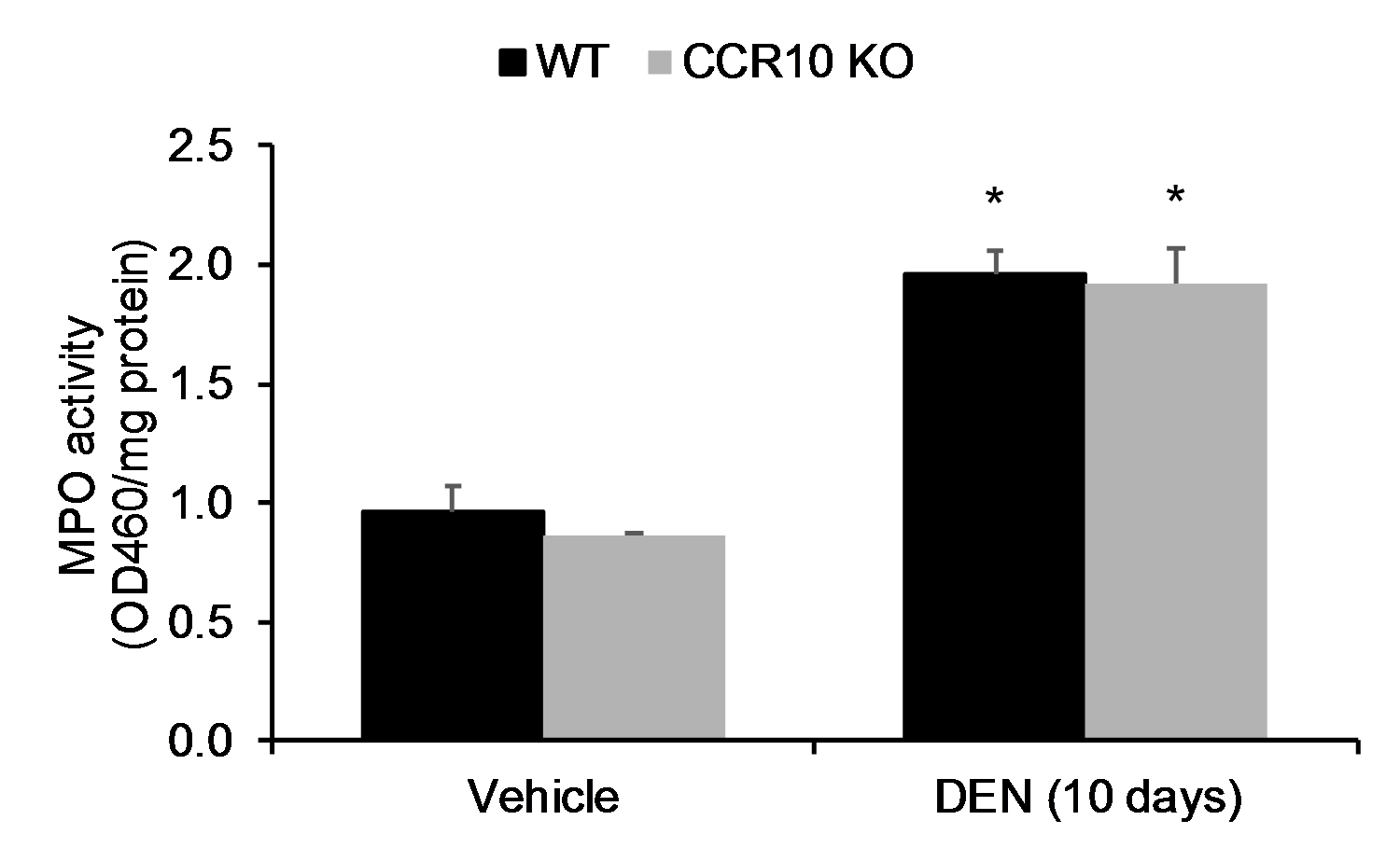

Supplement: Supplementary file 6 — Supplementary Figure 6 [file 41419_2018_267_MOESM6_ESM.tif]
