# Supplementary material for: The chemokine receptor CCR10 promotes inflammation-driven hepatocarcinogenesis via PI3K/Akt pathway activation
Source: Cell Death Dis. 2018 Feb 14;9(2):232. doi: 10.1038/s41419-018-0267-9 (PMC5833857; doi:10.1038/s41419-018-0267-9)
Supplement: Supplementary file 7 — Supplementary Information [file 41419_2018_267_MOESM7_ESM.docx]

**SUPPLEMENTARY INFORMATION**

**SUPPLEMENTARY TABLES**

**Supplementary Table 1. Demographic and Clinical Characteristics of Included Patients**

| **Parameter** | **HBV-infected HCC patients (n=81)** | **Normal patients (n=28)** |
| --- | --- | --- |
| Mean age | 51.9 ± 13.1 | 46.8 ± 11.6 |
| Sex (male %) | 86.4% | 46.4% |
| HBeAg (+) (%) | 23.5% | 0.0% |
| HBeAb (+) (%) | 65.4% | 39.3% |
| HBcAb (+) (%) | 90.1% | 96.4% |
| HBV-cAg S1 (+) (%) | 56.8% | 14.3% |
| HBV DNA | 10780 (500 – 182000) | 0 (0 - 0) |
| ALT, IU/l | 29.00 (21.00 – 46.00) | 21.00 (13.00 – 44.00) |
| TBil, µM | 12.43 (6.57 – 13.92) | 10.27 (6.69 – 11.32) |
| PT, s | 13.15 ± 1.39 | 12.33 ± 1.19 |
| ALB, g/l | 40.50 ± 4.63 | 45.21 ± 5.11 |
| AFP, ng/ml | 253.00 (19.00 – 2661.00) | 2.24 (1.42 – 3.10) |
| Liver cirrhosis G score | 1.83 ± 0.80 | - |
| Liver cirrhosis S score | 1.83 ± 0.76 | - |
| BCLC stage A (%) | 63.0% | - |
| BCLC stage B (%) | 19.8% | - |
| BCLC stage C (%) | 17.3% | - |

**Supplementary Table 2. Liver Function Testing of Wild-Type and CCR10-Knockout Mice**

| **Parameter** | **Wild-type**  **(CCR10+/+, n=12)** | **Heterozygous CCR10-Knockout (CCR10+/−, n=12)** | **Homozygous CCR10-Knockout (CCR10−/−, n=12)** |
| --- | --- | --- | --- |
| ALT, IU/l | 53.31 ± 5.24 | 50.15 ± 9.41 | 53.83 ± 5.95 |
| ALP, IU/l | 80.85 ± 8.13 | 78.22 ± 10.16 | 78.70 ± 8.73 |
| ALB, g/dl | 2.89 ± 0.38 | 2.91 ± 0.25 | 2.94 ± 0.35 |
| Total protein, g/dl | 5.15 ± 0.59 | 5.12 ± 0.62 | 5.15 ± 0.60 |

Results are reported as means ± standard deviations (SDs).

**Supplementary Table 3. Real-Time PCR Primer Sequences**

| **Gene** | **Primer sequences** |
| --- | --- |
| Human CCR10 | 5’-TGCTGGATACTGCCGATCTACTG-3’ (forward)  5’-TCTAGATTCGCAGCCCTAGTTGTC-3’ (reverse) |
| Murine CCR10 | 5’-AGAGCTCTGTTACAAGGCTGATGTC-3’ (forward)  5’-CAGGTGGTACTTCCTAGATTCCAGC-3’ (reverse) |
| Human CD45 | 5’-AGCCCTGCTTGTTGTTCTCT-3’ (forward)  5’-ACCCTGCATCTCCGTTTATC-3’ (reverse) |
| Murine CD45 | 5′-GCACCAGCTGATCTCCAGATA-3′ (forward)  5′-CAAACACCTACACCCAGT-3′ (reverse) |
| Human TNF | 5’-GTTCCTCAGCCTCTTCTCCT-3’ (forward)  5’-ACAACATGGGCTACAGGCTT-3’ (reverse) |
| Murine TNF | 5’-TTCTGTCTACTGAACTTCGGGGTGATCGGTCC-3’ (forward)  5’-GTATGAGATAGCAAATCGGCTGACGGTGTGGG-3’ (reverse) |
| Human IL-1α | 5’-AGATGCCTGAGATACCCAAAACC-3’ (forward)  5’-CCAAGCACACCCAGTAGTCT-3’ (reverse) |
| Murine IL-1α | 5’-CAAGATGGCCAAAGTTCGTGAC-3’ (forward)  5’-GTCTCATGAAGTGAGCCATAGC-3’ (reverse) |
| Human IL-1β | 5’-TCCCCAGCCCTTTTGTTGA-3’ (forward)  5’-TTAGAACCAAATGTGGCCGTG-3’ (reverse) |
| Murine IL-1β | 5’-ATGGCAACTGTTCCTGAACTCAACT-3’ (forward)  5’-CAGGACAGGTATAGATTCTTTCCTTT-3’ (reverse) |
| Human IL-6 | 5’-ATGTCTGAGGCTCATTCTGC-3’ (forward)  5’-GCGGCTACATCTTTGGAATC-3’ (reverse) |
| Murine IL-6 | 5’-AGGATACCACTCCCAACAGACCT-3’ (forward)  5’-CAAGTGCATCATCGTTGTTCATAC-3’ (reverse) |
| Human ICAM-1 | 5’-ATGCCCAGACATCTGTGTCC-3’ (forward)  5’-GGGGTCTCTATGCCCAACAA-3’ (reverse) |
| Murine ICAM-1 | 5’-GGCATTGTTCTCTAATGTCTCCG-3’ (forward)  5’-CCGCTCAGAAGAACCACCTTGG-3’ (reverse) |
| Human VCAM-1 | 5’-GGGAAGATGGTCGTGATCCTT-3’ (forward)  5’-TCTGGGGTGGTCTCGATTTTA-3’ (reverse) |
| Murine VCAM-1 | 5’-TACTCCCGTCATTGAGGATATTGG-3’ (forward)  5’-CTCCTTCACACACATAGACTCC-3’ (reverse) |
| Human CCL27 | 5’-CTCAGCTCTACCGAAAGCC-3’ (forward)  5’-GCCCATTTTCCTTAGCAT CC-3’ (reverse) |
| Human CCL28 | 5’-TGCACGGAG GTTTCACATCAT-3’ (forward)  5’-TTGGCAGCTTGCACTTTCATC-3’ (reverse) |
| Human β-actin | 5’-GTCTTCCCCTCCATCGTG-3’ (forward)  5’-AGGGTGAGGATGCCTCTCTT-3’ (reverse) |
| Murine β-actin | 5’-GGCTGTATTCCCCTCCATCG-3’ (forward)  5’-CCAGTTGGTAACAATGCCATGT-3’ (reverse) |

**SUPPLEMENTARY FIGURE LEGENDS**

**Supplementary Figure 1. Construction of CCR10-Knockout/EGFP Knockin Mice**

(A) Summary schematic of the gene targeting and screening approaches for constructing CCR10-KO/EGFP-knockin mice. (B) PCR screening for CCR10-KO embryonic stem (ES) cell clones with the P1 and P2 primers (see panel A; P1 and P2), which amplify a 0.8-Kb band from the CCR10-KO allele (but not the wild-type CCR10 allele or the genome-inserted CCR10-KO construct). (C) Southern blotting of CCR10-KO ES cell clones or mice. DNA was digested with SacI and then probed with a 0.8-Kb fragment localized upstream of CCR10’s 5′ arm (see panel A; 5′ probe). Southern blotting revealed bands for the wild-type (6.9-Kb band), neo-deleted targeted CCR10 allele (neo−, 7.8-Kb band), and neo-positive targeted CCR10 allele (neo+, 10-Kb band). (D) PCR-based identification of homozygous and heterozygous CCR10-KO mice with the P3, P4, and P5 primers (see panel A; P3, P4, and P5), which amplify a 330-bp wild-type fragment and a 280-bp knockout fragment. (E) Normal hepatocyte progenitor development in fetal livers of CCR10-KO mice. FACS-sorted fetal liver cells stained positive for the hepatocyte progenitor markers CD117 and CD34. (F) FACS validation of EGFP expression on gated CD117+/CD34+ hepatocyte progenitor cells from heterozygous CCR10-KO (CCR10+/−) mice and homozygous CCR10-KO (CCR10−/−) mice. ES cell clones (n=5 of each genotype) and mice (n=5 of each genotype) were analyzed. Abbreviations: +/+, wild-type ES cells or mice; neo+, CCR10-KO ES cells with inserted neo cassette; neo−/neo−, homozygous CCR10-KO/EGFP-knockin mice with neo cassette deleted; +/neo−, heterozygous CCR10-KO/EGFP-knockin mice with neo cassette deleted; S, SacI.

**Supplementary Figure 2. CD45 Transcript Assay to Detect Immune Cell Contamination among Isolated Hepatocyte Populations**

Hepatocytes were stringently isolated using a two-step collagenase isolation procedure followed by fluorescence-activated cell sorting (FACS, CD45-/CD95+ cell selection). Then, real-time RT-PCR of CD45 transcript expression was used to rule out immune cell contamination. (A) Real-time RT-PCR of CD45 mRNA expression in hepatocytes isolated from HCC tumor specimens and matching paracancerous tissue specimens as well as normal liver specimens. The positive control was total HCC tumor specimen tissue. (B) Real-time RT-PCR of CD45 mRNA expression in isolated hepatocytes from WT murine liver tissue after i.p. injection regimen of either CCl_4_ or physiological saline (vehicle), which revealed no discernable immune cell contamination. The positive control was total CCl_4_-treated liver tissue. (C) Real-time RT-PCR of CD45 mRNA expression in isolated hepatocytes from WT murine liver tissue after i.p. injection regimen of either DEN or physiological saline (vehicle), which revealed no discernable immune cell contamination. The positive control was total DEN-treated liver tissue. (D) Real-time RT-PCR of CD45 mRNA expression in isolated hepatocytes from murine liver tissue six hours after intraperitoneal (i.p.) injection of TNF, which revealed no discernable immune cell contamination. The positive control was total TNF-treated liver tissue.

**Supplementary Figure 3. Fluorescence-Activated Cell Sorting (FACS) Analysis of CCR10 Expression in Gated Human Hepatocytes**

(A) After two-step collagenase hepatocyte isolation, cell enrichment within the hepatocyte gate (high side scatter) is observable. (B) The isolated hepatocyte preparation is enriched for CD45-negative/CD95-positive hepatocytes. (D) After sorting for CD45-negative/CD95-positive hepatocytes, normal isolated hepatocytes show negligible CCR10 expression (black lines), while isolated hepatocytes from paracancerous and HCC tissues (red and blue lines, respectively) display positive CCR10 expression.

**Supplementary Figure 4. TNF Promotes Hepatocellular CCL28 Expression and Secretion in a Dose-Dependent Manner**

(A) Real-time RT-PCR analysis of CCL28 transcript expression in HepG2 and LO2 cells tissue four hours after treatment with various doses of TNF. (B) ELISA of CCL28 secretion from HepG2 and LO2 cells four hours after treatment with various doses of TNF. **P*<0.05 versus 0 ng/ml group, †*P*<0.05 versus 10 ng/ml group, ‡*P*<0.05 versus 20 ng/ml group. All values are reported as means ± standard errors of the mean (SEMs). n=12 mice in each group.

**Supplementary Figure 5. CCR10 Promotes Xenograft HCC Tumor Growth**

Hep3B cells were transfected with either CCR10-HA or vector-HA prior to transplantation in athymic nude mice. (A) Tumor volume growth curve of the Hep3B xenograft tumors over 24 days. (B) Macroscopic images of vector-HA-transfected Hep3B xenograft tumors (top) and CCR10-HA-transfected Hep3B xenograft tumors (bottom) after 24 days. Scale bar, 5 mm. (C) Hep3B xenograft tumor weights from the two experimental groups after 24 days. **P*<0.05 versus vector group. All values are reported as means ± standard deviations (SDs). n=10 mice in each group.

**Supplementary Figure 6. Knocking-Out CCR10 Does Not Affect Hepatic Neutrophilic Infiltration**

Livers were collected from CCR10 knockout (KO) and wild-type (WT) mice ten days after intraperitoneal (i.p.) injection of DEN or physiological saline (vehicle). Hepatic MPO activity was assayed via spectrophotometry. **P*<0.05 versus vehicle WT group, #*P*<0.05 versus DEN-treated WT group. All values are reported as means ± standard deviations (SDs). n=12 mice in each group.
